# Accelerating *L*_1_-penalized expectation maximization algorithm for latent variable selection in multidimensional two-parameter logistic models

**DOI:** 10.1371/journal.pone.0279918

**Published:** 2023-01-17

**Authors:** Laixu Shang, Ping-Feng Xu, Na Shan, Man-Lai Tang, George To-Sum Ho

**Affiliations:** 1 School of Mathematics and Statistics, Changchun University of Technology, Changchun, China; 2 Academy for Advanced Interdisciplinary Studies, Northeast Normal University, Changchun, China; 3 School of Psychology & Key Laboratory of Applied Statistics of MOE, Northeast Normal University, Changchun, China; 4 Department of Physics, Astronomy and Mathematics, School of Physics, Engineering & Computer Science, University of Hertfordshire, Hertfordshire, United Kingdom; 5 Department of Supply Chain and Information Management, Hang Seng University of Hong Kong, Hong Kong, China; Semnan University, IRAN, ISLAMIC REPUBLIC OF

## Abstract

One of the main concerns in multidimensional item response theory (MIRT) is to detect the relationship between observed items and latent traits, which is typically addressed by the exploratory analysis and factor rotation techniques. Recently, an EM-based *L*_1_-penalized log-likelihood method (EML1) is proposed as a vital alternative to factor rotation. Based on the observed test response data, EML1 can yield a sparse and interpretable estimate of the loading matrix. However, EML1 suffers from high computational burden. In this paper, we consider the coordinate descent algorithm to optimize a new weighted log-likelihood, and consequently propose an improved EML1 (IEML1) which is more than 30 times faster than EML1. The performance of IEML1 is evaluated through simulation studies and an application on a real data set related to the Eysenck Personality Questionnaire is used to demonstrate our methodologies.

## 1 Introduction

Multidimensional item response theory (MIRT) models are widely used to describe the relationship between the designed items and the intrinsic latent traits in psychological and educational tests [1]. Early researches for the estimation of MIRT models are confirmatory, where the relationship between the responses and the latent traits are pre-specified by prior knowledge [2, 3]. Under this setting, parameters are estimated by various methods including marginal maximum likelihood method [4] and Bayesian estimation [5]. However, misspecification of the item-trait relationships in the confirmatory analysis may lead to serious model lack of fit, and consequently, erroneous assessment [6].

To avoid the misfit problem caused by improperly specifying the item-trait relationships, the exploratory item factor analysis (IFA) [4, 7] is usually adopted. The exploratory IFA freely estimate the entire item-trait relationships (i.e., the loading matrix) only with some constraints on the covariance of the latent traits. To obtain a simpler loading structure for better interpretation, the factor rotation [8, 9] is adopted, followed by a cut-off. Although the exploratory IFA and rotation techniques are very useful, they can not be utilized without limitations. For some applications, different rotation techniques yield very different or even conflicting loading matrices. Therefore, it can be arduous to select an appropriate rotation or decide which rotation is the best [10]. In addition, different subjective choices of the cut-off value possibly lead to a substantial change in the loading matrix [11].

Recently, regularization has been proposed as a viable alternative to factor rotation, and it can automatically rotate the factors to produce a sparse loadings structure for exploratory IFA [12, 13]. Scharf and Nestler [14] compared factor rotation and regularization in recovering predefined factor loading patterns and concluded that regularization is a suitable alternative to factor rotation for psychometric applications. Regularization has also been applied to produce sparse and more interpretable estimations in many other psychometric fields such as exploratory linear factor analysis [11, 15, 16], the cognitive diagnostic models [17, 18], structural equation modeling [19], and differential item functioning analysis [20, 21].

For MIRT models, Sun et al. [12] proposed a latent variable selection framework to investigate the item-trait relationships by maximizing the *L*_1_-penalized likelihood [22]. In this framework, one can impose prior knowledge of the item-trait relationships into the estimate of loading matrix to resolve the rotational indeterminacy. Based on the observed test response data, the *L*_1_-penalized likelihood approach can yield a sparse loading structure by shrinking some loadings towards zero if the corresponding latent traits are not associated with a test item. Consequently, it produces a sparse and interpretable estimation of loading matrix, and it addresses the subjectivity of rotation approach.

Since the marginal likelihood for MIRT involves an integral of unobserved latent variables, Sun et al. [12] carried out the expectation maximization (EM) algorithm [23] to solve the *L*_1_-penalized optimization problem. We denote this method as EML1 for simplicity. In the E-step of EML1, numerical quadrature by fixed grid points is used to approximate the conditional expectation of the log-likelihood. This results in a naive weighted log-likelihood on augmented data set with size equal to *N* × *G*, where *N* is the total number of subjects and *G* is the number of grid points. To optimize the naive weighted *L*_1_-penalized log-likelihood in the M-step, the coordinate descent algorithm [24] is used, whose computational complexity is *O*(*N* × *G*). However, *N* × *G* is usually very large, and this consequently leads to high computational burden of the coordinate decent algorithm in the M-step. As shown by Sun et al. [12], EML1 requires several hours for MIRT models with three to four latent traits. Another limitation for EML1 is that it does not update the covariance matrix Σ of latent traits in the EM iteration. Sun et al. [12] proposed a two-stage method. It first computes an estimation of Σ via a constrained exploratory analysis under identification conditions, and then substitutes the estimated Σ into EML1 as a known Σ to estimate discrimination and difficulty parameters. However, our simulation studies show that the estimation of Σ obtained by the two-stage method could be quite inaccurate.

Further development for latent variable selection in MIRT models can be found in [25, 26]. Zhang and Chen [25] proposed a stochastic proximal algorithm for optimizing the *L*_1_-penalized marginal likelihood. They used the stochastic approximation in the stochastic step, which avoids repeatedly evaluating the numerical integral with respect to the multiple latent traits. However, the choice of several tuning parameters, such as a sequence of step size to ensure convergence and burn-in size, may affect the empirical performance of stochastic proximal algorithm. Xu et al. [26] applied the expectation model selection (EMS) algorithm [27] to minimize the *L*_0_-penalized log-likelihood (for example, the Bayesian information criterion [28]) for latent variable selection in MIRT models. In their EMS framework, the model (i.e., structure of loading matrix) and parameters (i.e., item parameters and the covariance matrix of latent traits) are updated simultaneously in each iteration. In the simulation of Xu et al. [26], the EMS algorithm runs significantly faster than EML1, but it still requires about one hour for MIRT with four latent traits.

In this paper, we focus on the classic EM framework of Sun et al. [12] and give an improved EM-based *L*_1_-penalized marginal likelihood (IEML1) with the M-step’s computational complexity being reduced to *O*(2 × *G*). The fundamental idea comes from the “artificial data” widely used in the EM algorithm for computing maximum marginal likelihood estimation in the IRT literature [4, 29–32]. In Bock and Aitkin (1981) [29] and Bock et al. (1988) [4], “artificial data” are the expected number of attempts and correct responses to each item in a sample of size *N* at a given ability level. Essentially, “artificial data” are used to replace the unobservable statistics in the expected likelihood equation of MIRT models. It should be noted that, the number of “artificial data” is *G* but not *N* × *G*, as “artificial data” correspond to *G* ability levels (i.e., grid points in numerical quadrature). As a result, the number of data involved in the weighted log-likelihood obtained in E-step is reduced and the efficiency of the M-step is then improved.

In our IEML1, we use a slightly different artificial data to obtain the weighted complete data log-likelihood [33] which is widely used in generalized linear models with incomplete data. Specifically, we classify the *N* × *G* augmented data into 2 × *G* artificial data (*z*, ***θ***^(*g*)^), where *z* (equals to 0 or 1) is the response to one item and ***θ***^(*g*)^ is one discrete ability level (i.e., grid point value). Thus, we obtain a new weighted *L*_1_-penalized log-likelihood based on a total number of 2 × *G* artificial data (*z*, ***θ***^(*g*)^), which reduces the computational complexity of the M-step to *O*(2 × *G*) from *O*(*N* × *G*).

In addition, it is crucial to choose the grid points being used in the numerical quadrature of the E-step for both EML1 and IEML1. There are various papers that discuss this issue in non-penalized maximum marginal likelihood estimation in MIRT models [4, 29, 30, 34]. To the best of our knowledge, there is however no discussion about the penalized log-likelihood estimator in the literature. In this paper, we will give a heuristic approach to choose artificial data with larger weights in the new weighted log-likelihood. Based on this heuristic approach, IEML1 needs only a few minutes for MIRT models with five latent traits.

The rest of the article is organized as follows. In Section 2, we introduce the multidimensional two-parameter logistic (M2PL) model as a widely used MIRT model, and review the *L*_1_-penalized log-likelihood method for latent variable selection in M2PL models. In Section 3, we give an improved EM-based *L*_1_-penalized log-likelihood method for M2PL models with unknown covariance of latent traits. In Section 4, we conduct simulation studies to compare the performance of IEML1, EML1, the two-stage method [12], a constrained exploratory IFA with hard-threshold (EIFAthr) and a constrained exploratory IFA with optimal threshold (EIFAopt). In Section 5, we apply IEML1 to a real dataset from the Eysenck Personality Questionnaire. A concluding remark is provided in Section 6.

## 2 Latent variable selection in multidimensional two-parameter logistic models

In this section, the M2PL model that is widely used in MIRT is introduced. Furthermore, the *L*_1_-penalized log-likelihood method for latent variable selection in M2PL models is reviewed.

### 2.1 Multidimensional two-parameter logistic model

Consider a *J*-item test that measures *K* latent traits of *N* subjects. Let ***Y*** = (*y*_*ij*_)_*N*×*J*_ be the dichotomous observed responses to the *J* items for all *N* subjects, where *y*_*ij*_ = 1 represents the correct response of subject *i* to item *j*, and *y*_*ij*_ = 0 represents the wrong response. Let ***θ***_*i*_ = (*θ*_*i*1_, …, *θ*_*iK*_)^*T*^ be the *K*-dimensional latent traits to be measured for subject *i* = 1, …, *N*. The relationship between the *j*th item response and the *K*-dimensional latent traits for subject *i* can be expressed by the M2PL model as follows

$$\begin{array}{c} F_{j}\left(\theta_{i}\right)\equiv P\left(y_{ij}=1|\theta_{i},a_{j},b_{j}\right)=\frac{\text{exp}(a_{j}^{T}\theta_{i}+b_{j})}{1+\text{exp}(a_{j}^{T}\theta_{i}+b_{j})}, \end{array}$$ (1)

where ***a***_*j*_ = (*a*_*j*1_, …, *a*_*jK*_)^*T*^ and *b*_*j*_ are known as the discrimination and difficulty parameters, respectively. The parameter *a*_*jk*_ ≠ 0 implies that item *j* is associated with latent trait *k*. *P*(*y*_*ij*_ = 1|***θ***_*i*_, ***a***_*j*_, *b*_*j*_) denotes the probability that subject *i* correctly responds to the *j*th item based on his/her latent traits ***θ***_*i*_ and item parameters ***a***_*j*_ and *b*_*j*_. For the sake of simplicity, we use the notation ***A*** = (***a***_1_, …, ***a***_*J*_)^*T*^, ***b*** = (*b*_1_, …, *b*_*J*_)^*T*^, and **Θ** = (***θ***_1_, …, ***θ***_*N*_)^*T*^. The discrimination parameter matrix ***A*** is also known as the loading matrix, and the corresponding structure is denoted by Λ = (λ_*jk*_) with λ_*jk*_ = *I*(*a*_*jk*_ ≠ 0).

In M2PL models, several general assumptions are adopted. The latent traits ***θ***_*i*_, *i* = 1, …, *N*, are assumed to be independent and identically distributed, and follow a *K*-dimensional normal distribution *N*(0, Σ) with zero mean vector and covariance matrix Σ = (*σ*_*kk*′_)_*K*×*K*_. Furthermore, the local independence assumption is assumed, that is, given the latent traits ***θ***_*i*_, *y*_*i*1_, …, *y*_*iJ*_ are conditional independent.

To guarantee the parameter identification and resolve the rotational indeterminacy for M2PL models, some constraints should be imposed. To identify the scale of the latent traits, we assume the variances of all latent trait are unity, i.e., *σ*_*kk*_ = 1 for *k* = 1, …, *K*. Dealing with the rotational indeterminacy issue requires additional constraints on the loading matrix ***A***. We adopt the constraints used by Sun et al. [12] and Xu et al. [26], that is, each of the first *K* items is associated with only one latent trait separately, i.e., *a*_*jj*_ ≠ 0 and *a*_*jk*_ = 0 for 1 ≤ *j* ≠ *k* ≤ *K*. In practice, the constraint on ***A*** should be determined according to priori knowledge of the item and the entire study.

### 2.2 Latent variable selection based on *L*_1_-penalized method

The response function for M2PL model in Eq (1) takes a logistic regression form, where *y*_*ij*_ acts as the response, the latent traits ***θ***_*i*_ as the covariates, ***a***_*j*_ and *b*_*j*_ as the regression coefficients and intercept, respectively. We are interested in exploring the subset of the latent traits related to each item, that is, to find all non-zero *a*_*jk*_s. This can be viewed as variable selection problem in a statistical sense.

Under the local independence assumption, the likelihood function of the complete data (***Y***, **Θ**) for M2PL model can be expressed as follow

$$\begin{array}{c} L\left(A,b,\Sigma |Y,\Theta \right)=\prod_{i=1}^{N}\varphi \left(\theta_{i}|\Sigma \right)\prod_{j=1}^{J}F_{j}{\left(\theta_{i}\right)}^{y_{ij}}{\left[1-F_{j}\left(\theta_{i}\right)\right]}^{1-y_{ij}}, \end{array}$$ (2)

where *φ*(***θ***_*i*_|Σ) is the density function of latent trait ***θ***_*i*_. The log-likelihood function of observed data ***Y*** can be written as

$$\begin{array}{c} l\left(A,b,\Sigma |Y\right)=\sum_{i=1}^{N}\text{log}[\int_{\theta_{i}}\varphi \left(\theta_{i}|\Sigma \right)\prod_{j=1}^{J}F_{j}{\left(\theta_{i}\right)}^{y_{ij}}{\left[1-F_{j}\left(\theta_{i}\right)\right]}^{1-y_{ij}}d\theta_{i}]. \end{array}$$ (3)

To investigate the item-trait relationships, Sun et al. [12] applied the *L*_1_-penalized marginal log-likelihood method to obtain the sparse estimate of ***A*** for latent variable selection in M2PL model. They carried out the EM algorithm [23] with coordinate descent algorithm [24] to solve the *L*_1_-penalized optimization problem. However, the covariance matrix Σ of latent traits is assumed to be known and is not realistic in real-world applications.

Instead, we will treat Σ as an unknown parameter and update it in each EM iteration. For this purpose, the *L*_1_-penalized optimization problem including Σ is represented as

$$\begin{array}{c} \left({\hat{A}}_{\eta },{\hat{b}}_{\eta },{\hat{\Sigma }}_{\eta }\right)=\underset{A,b,\Sigma }{\text{arg max}}\;l\left(A,b,\Sigma |Y\right)-{\eta ||A||}_{1} \end{array}$$ (4)

where $\left||A|{|}_{1}=\sum_{j=1}^{J}\sum_{k=1}^{K}|a_{jk}\right|$ denotes the entry-wise *L*_1_ norm of ***A***. The tuning parameter *η* > 0 controls the sparsity of ***A***. Larger value of *η* results in a more sparse estimate of ***A***. The tuning parameter is always chosen by cross validation or certain information criteria. In this paper, we employ the Bayesian information criterion (BIC) as described by Sun et al. [12].

## 3 Implementation of the EM algorithm

Due to the presence of the unobserved variable (e.g., the latent traits **Θ**), the parameter estimates in Eq (4) can not be directly obtained. Sun et al. [12] carried out EML1 to optimize Eq (4) with a known Σ. Similarly, we first give a naive implementation of the EM algorithm to optimize Eq (4) with an unknown Σ. Then, we give an efficient implementation with the M-step’s computational complexity being reduced to *O*(2 × *G*), where *G* is the number of grid points. Lastly, we will give a heuristic approach to choose grid points being used in the numerical quadrature in the E-step.

### 3.1 A naive implementation of the EM algorithm

The EM algorithm iteratively executes the expectation step (E-step) and maximization step (M-step) until certain convergence criterion is satisfied. Specifically, the E-step is to compute the *Q*-function, i.e., the conditional expectation of the *L*_1_-penalized complete log-likelihood with respect to the posterior distribution of latent traits **Θ**. The M-step is to maximize the *Q*-function. Let Ψ = (***A***, ***b***, Σ) be the set of model parameters, and Ψ^(*t*)^ = (***A***^(*t*)^, ***b***^(*t*)^, Σ^(*t*)^) be the parameters in the *t*th iteration. The (*t* + 1)th iteration is described as follows.

#### 3.1.1 E-step

In the E-step of the (*t* + 1)th iteration, under the current parameters Ψ^(*t*)^, we compute the *Q*-function involving a Σ-term as follows

$$\begin{array}{cc} Q(\Psi |\Psi^{\left(t\right)})= & E\left\{\text{log}\;L\left(\Psi |Y,\Theta \right)|Y,\Psi^{\left(t\right)}\right\}-{\eta ||A||}_{1}\\ = & Q_{0}\left(\Sigma |\Psi^{\left(t\right)}\right)+\sum_{j=1}^{J}Q_{j}\left(a_{j},b_{j}|\Psi^{\left(t\right)}\right), \end{array}$$ (5)

where *Q*_0_ is

$$Q_{0}\left(\Sigma |\Psi^{\left(t\right)}\right)=\sum_{i=1}^{N}E\left\{\text{log}\;\varphi \left(\theta_{i}|\Sigma \right)|y_{i},\Psi^{\left(t\right)}\right\}$$

and for *j* = 1, …, *J*, *Q*_*j*_ is

$$\begin{array}{cc} Q_{j}\left(a_{j},b_{j}|\Psi^{\left(t\right)}\right)\\ = & \sum_{i=1}^{N}E\left\{y_{ij}\;\text{log}\left(F_{j}\left(\theta_{i}\right)\right)+\left(1-y_{ij}\right)\text{log}\left(1-F_{j}\left(\theta_{i}\right)\right)|y_{i},\Psi^{\left(t\right)}\right\}-\eta ||a_{j}{\left|\right|}_{1}, \end{array}$$

where $\left||a_{j}|{|}_{1}=\sum_{k=1}^{K}|a_{jk}\right|$ denotes the *L*_1_-norm of vector ***a***_*j*_. The conditional expectations in *Q*_0_ and each *Q*_*j*_ are computed with respect to the posterior distribution of ***θ***_*i*_ as follows

$$p\left(\theta_{i}|y_{i},\Psi^{\left(t\right)}\right)\propto \prod_{j=1}^{J}{\left[F_{j}^{\left(t\right)}\left(\theta_{i}\right)\right]}^{y_{ij}}{\left[1-F_{j}^{\left(t\right)}\left(\theta_{i}\right)\right]}^{1-y_{ij}}\cdot \varphi \left(\theta_{i}|\Sigma^{\left(t\right)}\right),$$

where $F_{j}^{\left(t\right)}(\theta_{i})=P(y_{ij}=1|\theta_{i},a_{j}^{\left(t\right)},b_{j}^{\left(t\right)})$, $a_{j}^{\left(t\right)}$ is the *j*th row of ***A***^(*t*)^, and $b_{j}^{\left(t\right)}$ is the *j*th element in ***b***^(*t*)^.

Note that the conditional expectations in *Q*_0_ and each *Q*_*j*_ do not have closed-form solutions. It is usually approximated using the Gaussian-Hermite quadrature [4, 29] and Monte Carlo integration [35]. For simplicity, we approximate these conditional expectations by summations following Sun et al. [12]. Specifically, we choose fixed grid points $G\subseteq [-4,4{]}^{K}$ and the posterior distribution of ***θ***_*i*_ is then approximated by

$$\begin{array}{c} \tilde{p}\left(\theta_{i}|y_{i},\Psi^{\left(t\right)}\right)=\{\begin{array}{cc} C^{-1}\times \prod_{j=1}^{J}{\left[F_{j}^{\left(t\right)}\left(\theta_{i}\right)\right]}^{y_{ij}}{\left[1-F_{j}^{\left(t\right)}\left(\theta_{i}\right)\right]}^{1-y_{ij}}\cdot \varphi \left(\theta_{i}|\Sigma^{\left(t\right)}\right) & \text{if}\;\;\theta_{i}\in G,\\ 0 & \text{otherwise}, \end{array} \end{array}$$ (6)

where $C=\sum_{\theta_{i}^{\prime }\in G}\prod_{j=1}^{J}[F_{j}^{\left(t\right)}(\theta_{i}^{\prime }){]}^{y_{ij}}[1-F_{j}^{\left(t\right)}(\theta_{i}^{\prime }){]}^{1-y_{ij}}\cdot \varphi (\theta_{i}^{\prime }|\Sigma^{\left(t\right)})$ serves as a normalizing factor. Thus, *Q*_0_ can be approximated by

$$\begin{array}{c} {\tilde{Q}}_{0}\left(\Sigma |\Psi^{\left(t\right)}\right)=\sum_{i=1}^{N}\sum_{\theta_{i}\in G}\text{log}\;\varphi \left(\theta_{i}|\Sigma \right)\cdot \tilde{p}\left(\theta_{i}|y_{i},\Psi^{\left(t\right)}\right) \end{array}$$ (7)

and *Q*_*j*_ for *j* = 1, …, *J* is approximated by

$$\begin{array}{c} \begin{array}{cc} {\tilde{Q}}_{j}(a_{j} & ,b_{j}\left|\Psi^{\left(t\right)}\right)\\ = & \sum_{i=1}^{N}\sum_{\theta_{i}\in G}\left[y_{ij}\;\text{log}\left(F_{j}\left(\theta_{i}\right)\right)+\left(1-y_{ij}\right)\text{log}\left(1-F_{j}\left(\theta_{i}\right)\right)\right]\cdot \tilde{p}\left(\theta_{i}|y_{i},\Psi^{\left(t\right)}\right)-\eta ||a_{j}{\left|\right|}_{1}. \end{array} \end{array}$$ (8)

Hence, the *Q*-function can be approximated by

$$\begin{array}{c} \tilde{Q}\left(\Psi |\Psi^{\left(t\right)}\right)={\tilde{Q}}_{0}\left(\Sigma |\Psi^{\left(t\right)}\right)+\sum_{j=1}^{J}{\tilde{Q}}_{j}\left(a_{j},b_{j}|\Psi^{\left(t\right)}\right). \end{array}$$ (9)

#### 3.1.2 M-step

In the M-step of the (*t* + 1)th iteration, we maximize the approximation of *Q*-function obtained by E-step

$$\begin{array}{c} \Psi^{\left(t+1\right)}=\left(A^{\left(t+1\right)},b^{\left(t+1\right)},\Sigma^{\left(t+1\right)}\right)=\underset{\Psi }{\text{arg}\;\text{max}}\;\tilde{Q}\left(\Psi |\Psi^{\left(t\right)}\right), \end{array}$$ (10)

subject to Σ ≻ 0 and diag(Σ) = **1**, where Σ ≻ 0 denotes that Σ is a positive definite matrix, and diag(Σ) = **1** denotes that all the diagonal entries of Σ are unity.

It can be easily seen from Eq (9) that $\tilde{Q}$ can be factorized as the summation of ${\tilde{Q}}_{0}$ involving Σ and ${\tilde{Q}}_{j}$ involving (***a***_*j*_, *b*_*j*_). Thus, the maximization problem in Eq (10) can be decomposed to maximizing ${\tilde{Q}}_{0}$ and maximizing penalized ${\tilde{Q}}_{j}$ separately, that is,

$$\begin{array}{c} \Sigma^{\left(t+1\right)}=\underset{\Sigma }{\text{arg}\;\text{max}}\;{\tilde{Q}}_{0}\left(\Sigma |\Psi^{\left(t\right)}\right)\;\;\text{s.t.}\;\;\Sigma ≻0\;\;\text{and}\;\;\text{diag}\left(\Sigma \right)=1, \end{array}$$ (11)

and for *j* = 1, …, *J*,

$$\begin{array}{c} \left(a_{j}^{\left(t+1\right)},b_{j}^{\left(t+1\right)}\right)=\underset{a_{j},b_{j}}{\text{arg}\;\text{max}}\;{\tilde{Q}}_{j}\left(a_{j},b_{j}|\Psi^{\left(t\right)}\right), \end{array}$$ (12)

For maximization problem (11), ${\tilde{Q}}_{0}$ can be represented as

$${\tilde{Q}}_{0}\left(\Sigma |\Psi^{\left(t\right)}\right)=-\frac{1}{2}\left\{NK\text{log}\left(2\pi \right)+N\text{log}\;\text{det}\Sigma +N\text{tr}\left[\Sigma^{-1}S^{*}\right]\right\},$$

where tr[⋅] denotes the trace operator of a matrix, where

$$\begin{array}{c} S^{*}=N^{-1}\sum_{i=1}^{N}\sum_{\theta_{i}\in G}\theta_{i}\theta_{i}^{T}\cdot \tilde{p}\left(\theta_{i}|y_{i},\Psi^{\left(t\right)}\right). \end{array}$$ (13)

Therefore, the optimization problem in (11) is known as a semi-definite programming problem in convex optimization. We can obtain the Σ^(*t* + 1)^ in the same way as Zhang et al. [36] by applying a proximal gradient descent algorithm [37]. It is noteworthy that in the EM algorithm used by Sun et al. [12], *Q*_0_ is a constant and thus need not be optimized, as Σ is assumed to be known.

For maximization problem (12), it is noted that ${\tilde{Q}}_{j}$ in Eq (8) can be regarded as the weighted *L*_1_-penalized log-likelihood in logistic regression with naive augmented data (*y*_*ij*_, ***θ***_*i*_) and weights $\tilde{p}(\theta_{i}|y_{i},A^{\left(t\right)},b^{\left(t\right)})$, where $\theta_{i}\in G$. Hence, the maximization problem in (Eq 12) is equivalent to the variable selection in logistic regression based on the *L*_1_-penalized likelihood. Several existing methods such as the coordinate decent algorithm [24] can be directly used.

After solving the maximization problems in Eqs (11) and (12), it is straightforward to obtain the parameter estimates of Σ^(*t* + 1)^, $A^{\left(t+1\right)}=(a_{1}^{\left(t+1\right)},\ldots ,a_{J}^{\left(t+1\right)}{)}^{T}$ and $b^{\left(t+1\right)}=(b_{1}^{\left(t+1\right)},\ldots ,b_{J}^{\left(t+1\right)}{)}^{T}$ for the next iteration.

We call the implementation described in this subsection the naive version since the M-step suffers from a high computational burden. It should be noted that the computational complexity of the coordinate descent algorithm for maximization problem (12) in the M-step is proportional to the sample size of the data set used in the logistic regression [24]. In (12), the sample size (i.e., *N* × *G*) of the naive augmented data set {(*y*_*ij*_, ***θ***_*i*_)|*i* = 1, …, *N*, and $\theta_{i}\in G\}$ is usually large, where *G* is the number of quadrature grid points in G. For example, if *N* = 1000, *K* = 3 and 11 quadrature grid points are used in each latent trait dimension, then *G* = 1331 and *N* × *G* = 1.331 × 10^6^. This leads to a heavy computational burden for maximizing (12) in the M-step. As a result, the EML1 developed by Sun et al. [12] is computationally expensive.

### 3.2 An improved EM-based *L*_1_-penalized likelihood method

In this subsection, motivated by the idea about “artificial data” widely used in maximum marginal likelihood estimation in the IRT literature [30], we will derive another form of weighted log-likelihood based on a new artificial data set with size 2 × *G*. Therefore, the computational complexity of the M-step is reduced to *O*(2 × *G*) from *O*(*N* × *G*).

As described in Section 3.1.1, we use the same set G of fixed grid points for all ***θ***_*i*_s to approximate the conditional expectation. Let $G=\{\theta^{\left(g\right)},g=1,\ldots ,G\}$ with ***θ***^(*g*)^ representing a discrete ability level, and $\tilde{p}(\theta^{\left(g\right)}|y_{i},\Psi^{\left(t\right)})$ denote the value of $\tilde{p}(\theta_{i}|y_{i},\Psi^{\left(t\right)})$ at ***θ***_*i*_ = ***θ***^(*g*)^. Using the traditional “artificial data” described in Baker and Kim [30], we can write ${\tilde{Q}}_{j}$ as

$$\begin{array}{cc} & {\tilde{Q}}_{j}\left(a_{j},b_{j}|\Psi^{\left(t\right)}\right)\\ = & \sum_{i=1}^{N}\sum_{g=1}^{G}[y_{ij}\text{log}\left(F_{j}\left(\theta^{\left(g\right)}\right)\right)+\left(1-y_{ij}\right)\text{log}\left(1-F_{j}\left(\theta^{\left(g\right)}\right)\right)]\cdot \tilde{p}\left(\theta^{\left(g\right)}|y_{i},\Psi^{\left(t\right)}\right)-\eta ||a_{j}{\left|\right|}_{1}\\ = & \sum_{g=1}^{G}[\text{log}\left(F_{j}\left(\theta^{\left(g\right)}\right)\right)\cdot r_{jg}^{\left(t\right)}+\text{log}\left(1-F_{j}\left(\theta^{\left(g\right)}\right)\right)\cdot \left(f_{g}^{\left(t\right)}-r_{jg}^{\left(t\right)}\right)]-\eta ||a_{j}{\left|\right|}_{1}, \end{array}$$ (14)

where $f_{g}^{\left(t\right)}=\sum_{i=1}^{N}\tilde{p}(\theta^{\left(g\right)}|y_{i},\Psi^{\left(t\right)})$ is the “expected sample size” at ability level ***θ***^(*g*)^, and $r_{jg}^{\left(t\right)}=\sum_{i=1}^{N}y_{ij}\tilde{p}(\theta^{\left(g\right)}|y_{i},\Psi^{\left(t\right)})$ is the “expected frequency” of correct response to item *j* at ability ***θ***^(*g*)^. Note that, in the IRT literature, $f_{g}^{\left(t\right)}$ and $r_{jg}^{\left(t\right)}$ are known as “artificial data”, and they are applied to replace the unobservable sufficient statistics in the complete data likelihood equation in the E-step of the EM algorithm for computing maximum marginal likelihood estimation [30–32]. If *η* = 0, differentiating Eq (14), we can obtain a likelihood equation involving the traditional “artificial data”, which can be solved by standard optimization methods [30, 32].

For *L*_1_-penalized log-likelihood estimation, we should maximize Eq (14) for *η* > 0. Although the coordinate descent algorithm [24] can be applied to maximize Eq (14), some technical details are needed. In this paper, from a novel perspective, we will view ${\tilde{Q}}_{j}(a_{j},b_{j}|\Psi^{\left(t\right)})$ as a weighted *L*_1_-penalized log-likelihood of logistic regression based on our new artificial data inspirited by Ibrahim (1990) [33] and maximize ${\tilde{Q}}_{j}(a_{j},b_{j}|\Psi^{\left(t\right)})$ by applying the efficient R package glmnet [24].

Specifically, we group the *N* × *G* naive augmented data in Eq (8) into 2 × *G* new artificial data (*z*, ***θ***^(*g*)^), where *z* (equals to 0 or 1) is the response to item *j* and ***θ***^(*g*)^ is a discrete ability level. Thus, ${\tilde{Q}}_{j}$ in Eq (8) can be rewritten as

$$\begin{array}{cc} & {\tilde{Q}}_{j}\left(a_{j},b_{j}|\Psi^{\left(t\right)}\right)\\ = & \sum_{i=1}^{N}\sum_{g=1}^{G}\sum_{z=0,1}I\left(y_{ij}=z\right)\left[z\;\text{log}\left(F_{j}\left(\theta^{\left(g\right)}\right)\right)+\left(1-z\right)\text{log}\left(1-F_{j}\left(\theta^{\left(g\right)}\right)\right)\right]\cdot \tilde{p}\left(\theta^{\left(g\right)}|y_{i},\Psi^{\left(t\right)}\right)-\eta ||a_{j}{\left|\right|}_{1}\\ = & \sum_{g=1}^{G}\sum_{z=0,1}[z\;\text{log}\left(F_{j}\left(\theta^{\left(g\right)}\right)\right)+\left(1-z\right)\text{log}\left(1-F_{j}\left(\theta^{\left(g\right)}\right)\right)]\cdot \sum_{i=1}^{N}I\left(y_{ij}=z\right)\tilde{p}\left(\theta^{\left(g\right)}|y_{i},\Psi^{\left(t\right)}\right)-\eta ||a_{j}{\left|\right|}_{1}\\ = & \sum_{g=1}^{G}\sum_{z=0,1}[z\;\text{log}\left(F_{j}\left(\theta^{\left(g\right)}\right)\right)+\left(1-z\right)\text{log}\left(1-F_{j}\left(\theta^{\left(g\right)}\right)\right)]\cdot w_{j}^{\left(t\right)}\left(z,\theta^{\left(g\right)}\right)-\eta ||a_{j}{\left|\right|}_{1}, \end{array}$$ (15)

where $w_{j}^{\left(t\right)}(z,\theta^{\left(g\right)})=\sum_{i=1}^{N}I(y_{ij}=z)\tilde{p}(\theta^{\left(g\right)}|y_{i},\Psi^{\left(t\right)})$ is the “expected frequency” of correct or incorrect response to item *j* at ability ***θ***^(*g*)^. The second equality in Eq (15) holds since *z* and *F*_*j*_(***θ***^(*g*)^)) do not depend on *y*_*ij*_ and the order of the summation is interchanged. Thus, we obtain a new form of weighted *L*_1_-penalized log-likelihood of logistic regression in the last line of Eq (15) based on the new artificial data (*z*, ***θ***^(*g*)^) with a weight $w_{j}^{\left(t\right)}(z,\theta^{\left(g\right)})$. Note that $w_{j}^{\left(t\right)}(1,\theta^{\left(g\right)})=r_{jg}^{\left(t\right)}$ and $w_{j}^{\left(t\right)}(0,\theta^{\left(g\right)})=f_{g}^{\left(t\right)}-r_{jg}^{\left(t\right)}$, so the traditional “artificial data” can be viewed as weights for our new artificial data (*z*, ***θ***^(*g*)^).

Since Eq (15) is a weighted *L*_1_-penalized log-likelihood of logistic regression, it can be optimized directly via the efficient R package glmnet [24]. This is an advantage of using Eq (15) instead of Eq (14). Moreover, the size of the new artificial data set {(*z*, ***θ***^(*g*)^)|*z* = 0, 1, and $\theta^{\left(g\right)}\in G\}$ involved in Eq (15) is 2 × *G*, which is substantially smaller than *N* × *G*. This significantly reduces the computational burden for optimizing ${\tilde{Q}}_{j}$ in the M-step. We call this version of EM as the improved EML1 (IEML1). Since the computational complexity of the coordinate descent algorithm is *O*(*M*) where *M* is the sample size of data involved in penalized log-likelihood [24], the computational complexity of M-step of IEML1 is reduced to *O*(2 × *G*) from *O*(*N* × *G*).

It is noteworthy that, for ***y***_*i*_ = ***y***_*i*′_ with the same response pattern, the posterior distribution of ***θ***_*i*_ is the same as that of ***θ***_*i*′_, i.e., $\tilde{p}(\theta^{\left(g\right)}|y_{i},\Psi^{\left(t\right)})=\tilde{p}(\theta^{\left(g\right)}|y_{i^{\prime }},\Psi^{\left(t\right)})$. When the sample size *N* is large, the item response vectors ***y***_1_, ⋯, ***y***_*N*_ can be grouped into distinct response patterns, and then the summation in computing $w_{j}^{\left(t\right)}(z,\theta^{\left(g\right)})$ is not over *N*, but over the number of distinct patterns, which will greatly reduce the computational time [30].

It should be noted that any fixed quadrature grid points set, such as Gaussian-Hermite quadrature points set, will result in the same weighted *L*_1_-penalized log-likelihood as in Eq (15). However, neither the adaptive Gaussian-Hermite quadrature [34] nor the Monte Carlo integration [35] will result in Eq (15) since the adaptive Gaussian-Hermite quadrature requires different adaptive quadrature grid points for different ***θ***_*i*_ while the Monte Carlo integration usually draws different Monte Carlo samples for different ***θ***_*i*_.

### 3.3 Heuristic approach for choosing grid points

In the new weighted log-likelihood in Eq (15), the more artificial data (*z*, ***θ***^(*g*)^) are used, the more accurate the approximation of ${\tilde{Q}}_{j}$ is; but, the more computational burden IEML1 has. To reduce the computational burden of IEML1 without sacrificing too much accuracy, we will give a heuristic approach for choosing a few grid points used to compute ${\tilde{Q}}_{j}$.

Let us consider a motivating example based on a M2PL model with item discrimination parameter matrix ***A***_1_ with *K* = 3 and *J* = 40, which is given in Table A in S1 Appendix. The grid point set $G=S^{3}$, where S denotes a set of equally spaced 11 grid points on the interval [−4, 4]. Therefore, the size of our new artificial data set used in Eq (15) is 2 × 11^3^ = 2662. Based on one iteration of the EM algorithm for one simulated data set, we calculate the weights of the new artificial data $(z,\theta^{\left(g\right)})\in \{0,1\}\times G$ and then sort them in descending order.

**Fig 1:**
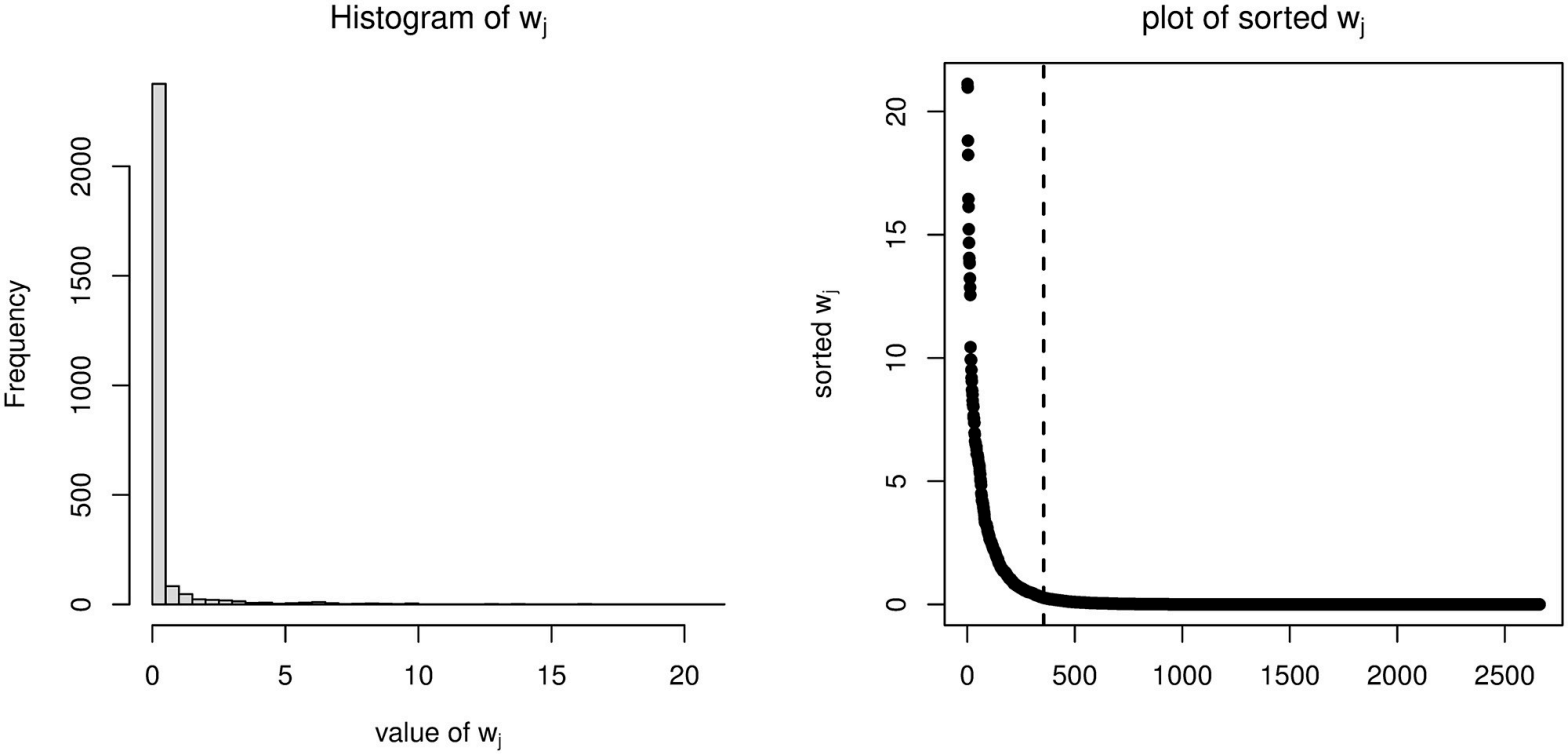
Histogram of *w*_*j*_ (left column) and plot of sorted *w*_*j*_ (right column).

**Fig 2:**
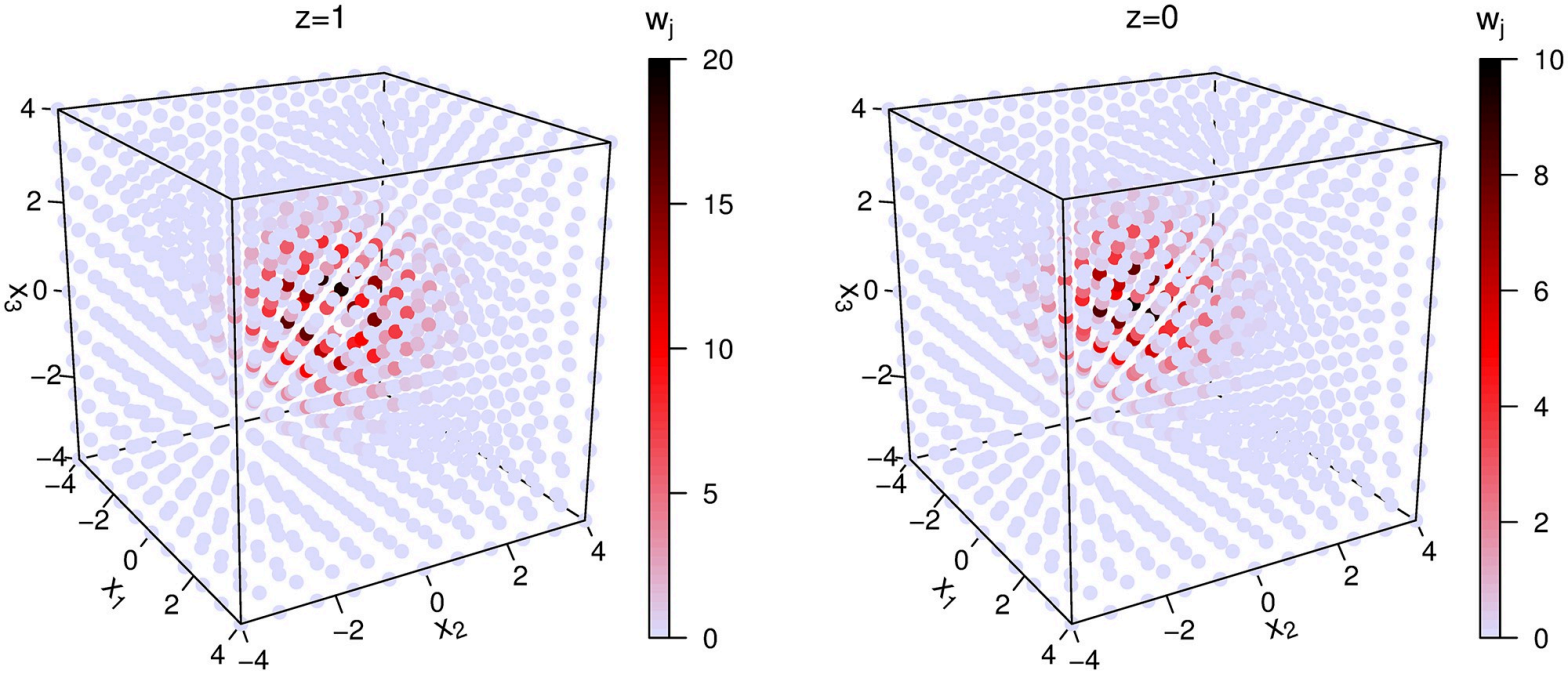
Scatter plots of the grid points with the weights *w*_*j*_ under *z* = 1 (left column) and *z* = 0 (right column).

Fig 1 (left) gives the histogram of all weights, which shows that most of the weights are very small and only a few of them are relatively large. Fig 1 (right) gives the plot of the sorted weights, in which the top 355 sorted weights are bounded by the dashed line. The sum of the top 355 weights consitutes 95.9% of the sum of all the 2662 weights. This suggests that only a few (*z*, ***θ***^(*g*)^) contribute significantly to ${\tilde{Q}}_{j}$. Furthermore, Fig 2 presents scatter plots of our artificial data (*z*, ***θ***^(*g*)^), in which the darker the color of (*z*, ***θ***^(*g*)^), the greater the weight $w_{j}^{\left(t\right)}(z,\theta^{\left(g\right)})$. It can be seen roughly that most (*z*, ***θ***^(*g*)^) with greater weights are included in {0, 1} × [−2.4, 2.4]^3^. In fact, artificial data with the top 355 sorted weights in Fig 1 (right) are all in {0, 1} × [−2.4, 2.4]^3^. These observations suggest that we should use a reduced grid point set $G^{*}$ with each dimension consisting of 7 equally spaced grid points on the interval [−2.4, 2.4]. Thus, the size of the corresponding reduced artificial data set is 2 × 7^3^ = 686. In this way, only 686 artificial data are required in the new weighted log-likelihood in Eq (15). Our simulation studies show that IEML1 with this reduced artificial data set performs well in terms of correctly selected latent variables and computing time.

In the literature, Xu et al. [26] gives a similar approach to choose the naive augmented data (*y*_*ij*_, ***θ***_*i*_) with larger weight $\tilde{p}(\theta_{i}|y_{i},\Psi^{\left(t\right)})$ for computing Eq (8). In this paper, we however choose our new artificial data (*z*, ***θ***^(*g*)^) with larger weight $w_{j}^{\left(t\right)}(z,\theta^{\left(g\right)})$ to compute Eq (15).

## 4 Simulation studies

In this section, we conduct simulation studies to evaluate and compare the performance of our IEML1, the EML1 proposed by Sun et al. [12] and the constrained exploratory IFAs with hard-threshold and optimal threshold. In all methods, we use the same identification constraints described in subsection 2.1 to resolve the rotational indeterminacy. In addition, we also give simulation studies to show the performance of the heuristic approach for choosing grid points. The R codes of the IEML1 method are provided in S4 Appendix.

Here, we consider three M2PL models with the item number *J* equal to 40. Three true discrimination parameter matrices ***A***_1_, ***A***_2_ and ***A***_3_ with *K* = 3, 4, 5 are shown in Tables A, C and E in S1 Appendix, respectively. The corresponding difficulty parameters ***b***_1_, ***b***_2_ and ***b***_3_ are listed in Tables B, D and F in S1 Appendix. The non-zero discrimination parameters are generated from the identically independent uniform distribution *U*(0.5, 2). The true difficulty parameters are generated from the standard normal distribution. The diagonal elements of the true covariance matrix Σ of the latent traits are setting to be unity with all off-diagonals being 0.1.

For parameter identification, we constrain items 1, 10, 19 to be related only to latent traits 1, 2, 3 respectively for *K* = 3, that is, (*a*_1_, *a*_10_, *a*_19_)^*T*^ in ***A***_1_ was fixed as diagonal matrix in each EM iteration. Similarly, items 1, 7, 13, 19 are related only to latent traits 1, 2, 3, 4 respectively for *K* = 4 and items 1, 5, 9, 13, 17 are related only to latent traits 1, 2, 3, 4, 5 respectively for *K* = 5.

Two sample size (i.e., *N* = 500, 1000) are considered. For each setting, we draw 100 independent data sets for each M2PL model. We obtain results by IEML1 and EML1 and evaluate their results in terms of computation efficiency, correct rate (CR) for the latent variable selection and accuracy of the parameter estimation. The computation efficiency is measured by the average CPU time over 100 independent runs. The CR for the latent variable selection is defined by the recovery of the loading structure Λ = (λ_*jk*_) as follows:

$$\text{CR}=\frac{1}{K(J-K)}\sum_{1\leq j\leq J,1\leq k\leq K,a_{jk}\;\;\text{is}\;\text{not}\;\text{fixed}\;\text{for}\;\text{identification}}I\left({\hat{\lambda }}_{jk}=\lambda_{jk}\right),$$

where $\hat{\Lambda }=({\hat{\lambda }}_{jk})$ is an estimate of the true loading structure Λ. The following mean squared error (MSE) is used to measure the accuracy of the parameter estimation:

$$\text{MSE}\left(a_{jk}\right)=\frac{1}{S}\sum_{s=1}^{S}{\left({\hat{a}}_{jk}^{s}-a_{jk}\right)}^{2},$$

where $a_{jk}^{s}$ denotes the estimate of *a*_*jk*_ from the *s*th replication and *S* = 100 is the number of data sets. The MSE of each *b*_*j*_ in ***b*** and *σ*_*kk*′_ in Σ is calculated similarly to that of *a*_*jk*_.

### 4.1 Computational efficiency

We first compare computational efficiency of IEML1 and EML1. To make a fair comparison, the covariance of latent traits Σ is assumed to be known for both methods in this subsection.

In this study, we consider M2PL with ***A***_1_. We use the fixed grid point set G=S×S×S, where S is the set of equally spaced 11 grid points on the interval [4, 4]. In each M-step, the maximization problem in (12) is solved by the R-package glmnet for both methods. Due to tedious computing time of EML1, we only run the two methods on 10 data sets. For each replication, the initial value of (***a***_1_, ***a***_10_, ***a***_19_)^*T*^ is set as identity matrix, and other initial values in ***A*** are set as 1/*J* = 0.025. The initial value of ***b*** is set as the zero vector. The candidate tuning parameters are given as (0.10, 0.09, …, 0.01) × *N*, and we choose the best tuning parameter by Bayesian information criterion as described by Sun et al. [12].

The average CPU time (in seconds) for IEML1 and EML1 are given in Table 1. From Table 1, IEML1 runs at least 30 times faster than EML1. Moreover, IEML1 and EML1 yield comparable results with the absolute error no more than 10^−13^. It numerically verifies that two methods are equivalent.

**Table 1:** The average CPU time in seconds for IEML1 and EML1 under *K* = 3 and *J* = 40.

	**IEML1**	**EML1**
*N* = 500	91	3019
*N* = 1000	114	6553

### 4.2 Simulation for the unknown Σ case

In this subsection, we compare our IEML1 with a two-stage method proposed by Sun et al. [12], a constrained exploratory IFA with hard threshold (EIFAthr) and a constrained exploratory IFA with optimal threshold (EIFAopt). In the EIFAthr, all parameters are estimated via a constrained exploratory analysis satisfying the identification conditions, and then the estimated discrimination parameters that smaller than a given threshold are truncated to be zero. In the simulation studies, several thresholds, i.e., 0.30, 0.35, …, 0.70, are used, and the corresponding EIFAthr are denoted by EIFA0.30, EIFA0.35, …, EIFA0.70, respectively. In EIFAthr, it is subjective to preset a threshold, while in EIFAopt we further choose the optimal truncated estimates correponding to the optimal threshold with minimum BIC value from several given thresholds (e.g., 0.30, 0.35, …, 0.70 used in EIFAthr) in a data-driven manner.

For IEML1, the initial value of Σ is set to be an identity matrix. For other three methods, a constrained exploratory IFA is adopted to estimate Σ first by R-package mirt with the setting being “method = EM” and the same grid points are set as in subsection 4.1.

**Fig 3:**
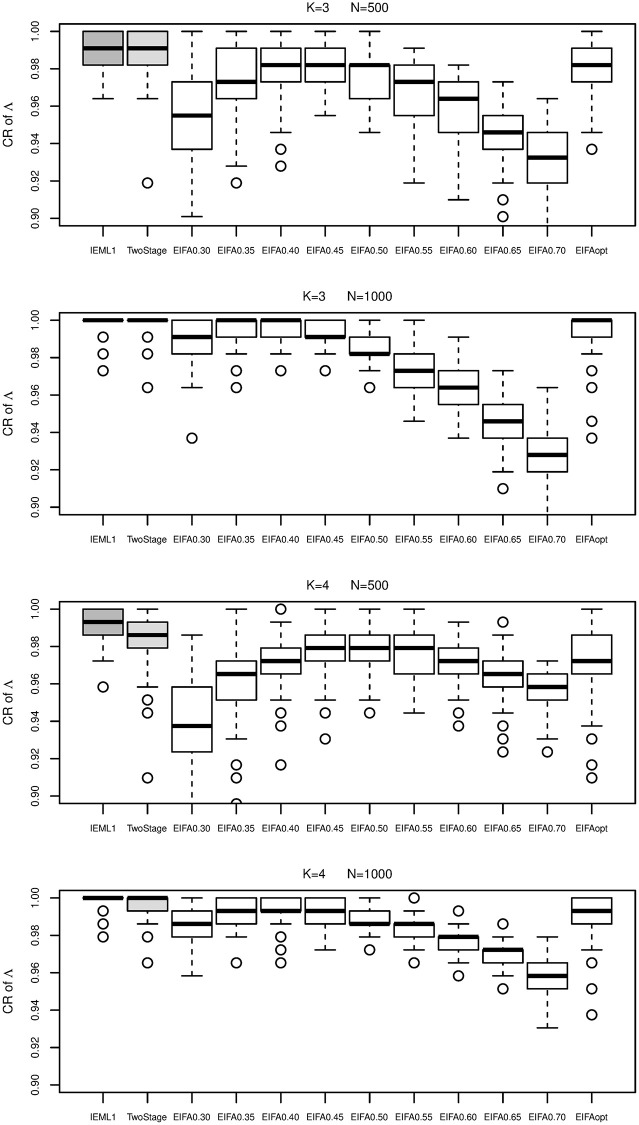
Boxplots of the correct rate of Λ obtained by IEML1 (dark gray boxes), two-stage (light gray boxes), EIFAthr and EIFAopt (white boxes) for *K* = 3 and 4 under sample size *N* = 500 and 1000.

We consider M2PL models with ***A***_1_ and ***A***_2_ in this study. To compare the latent variable selection performance of all methods, the boxplots of CR are dispalyed in Fig 3. From Fig 3, IEML1 performs the best and then followed by the two-stage method. As we expect, different hard thresholds leads to different estimates and the resulting different CR, and it would be difficult to choose a best hard threshold in practices. EIFAopt performs better than EIFAthr. As complements to CR, the false negative rate (FNR), false positive rate (FPR) and precision are reported in S2 Appendix. The boxplots of these metrics show that our IEML1 has very good performance overall.

**Fig 4:**
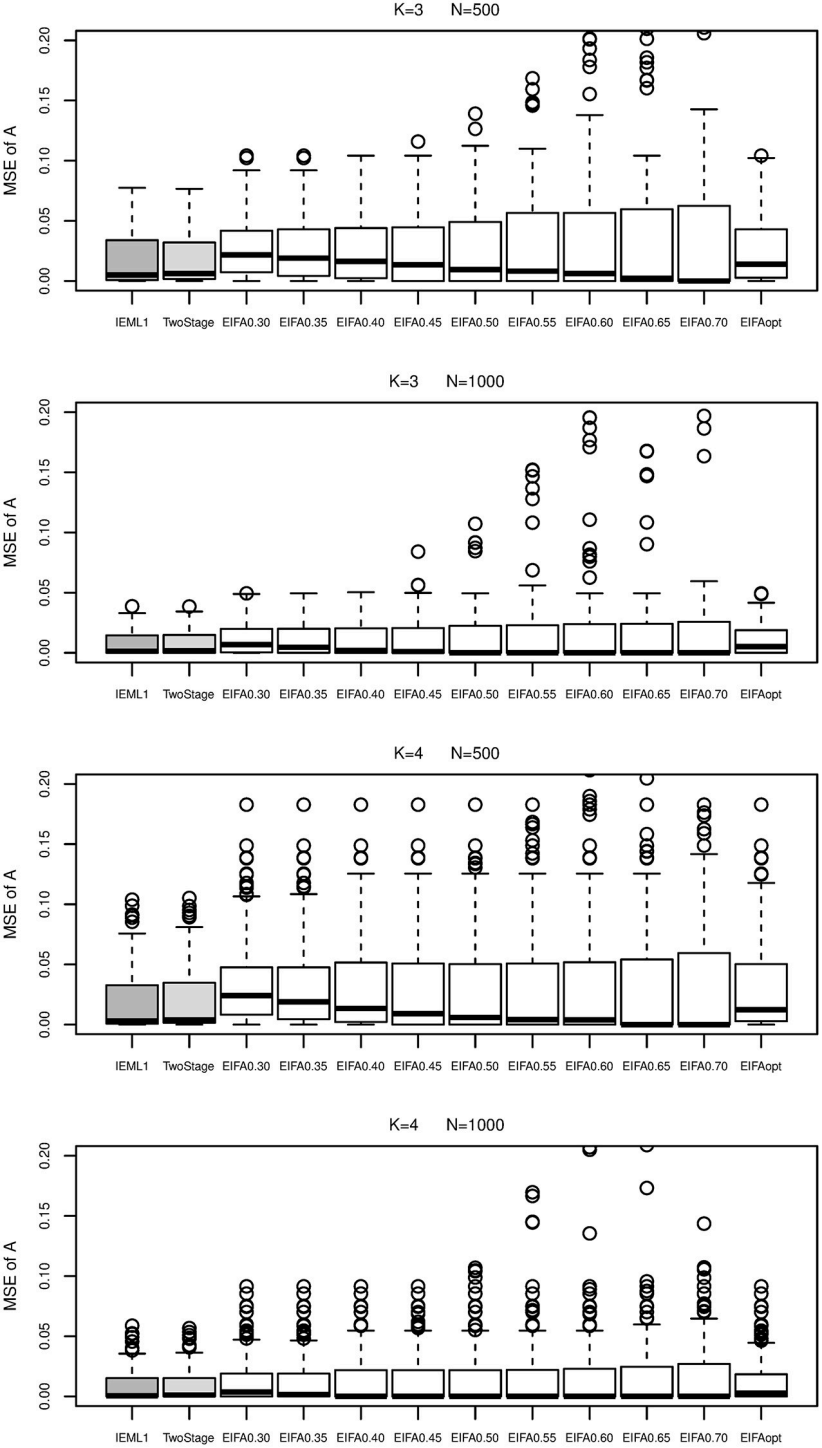
Boxplots of the MSE of *A* obtained by IEML1 (dark gray boxes), two-stage (light gray boxes), EIFAthr and EIFAopt (white boxes) for *K* = 3 and 4 under sample size *N* = 500 and 1000.

Fig 4 presents boxplots of the MSE of ***A*** obtained by all methods. From Fig 4, IEML1 and the two-stage method perform similarly, and better than EIFAthr and EIFAopt. We can see that larger threshold leads to smaller median of MSE, but some very large MSEs in EIFAthr.

**Fig 5:**
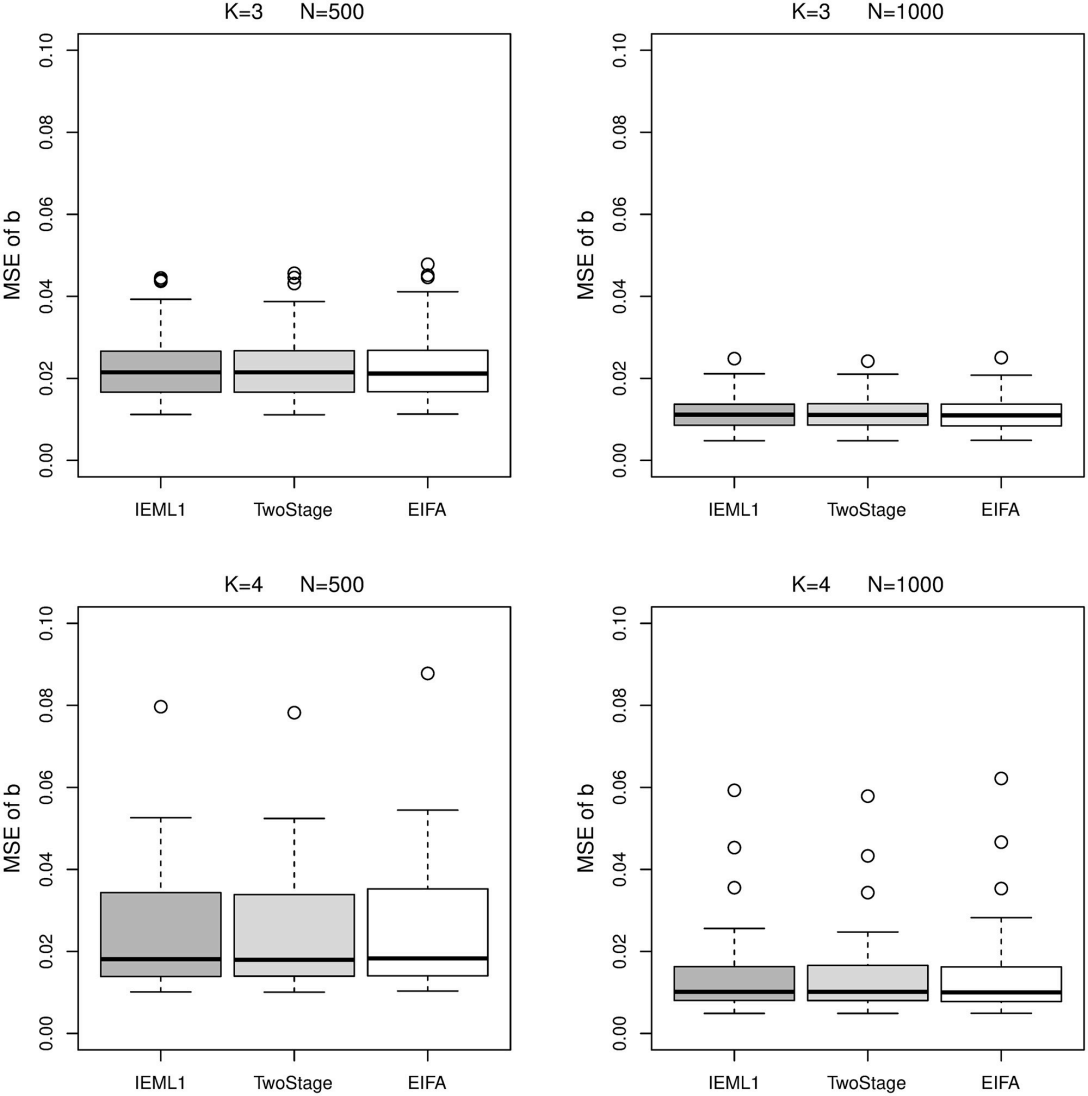
Boxplots of the MSE of *b* obtained by IEML1 (dark gray boxes), two-stage (light gray boxes), EIFAthr and EIFAopt (white boxes) for *K* = 3 and 4 under sample size *N* = 500 and 1000.

**Fig 6:**
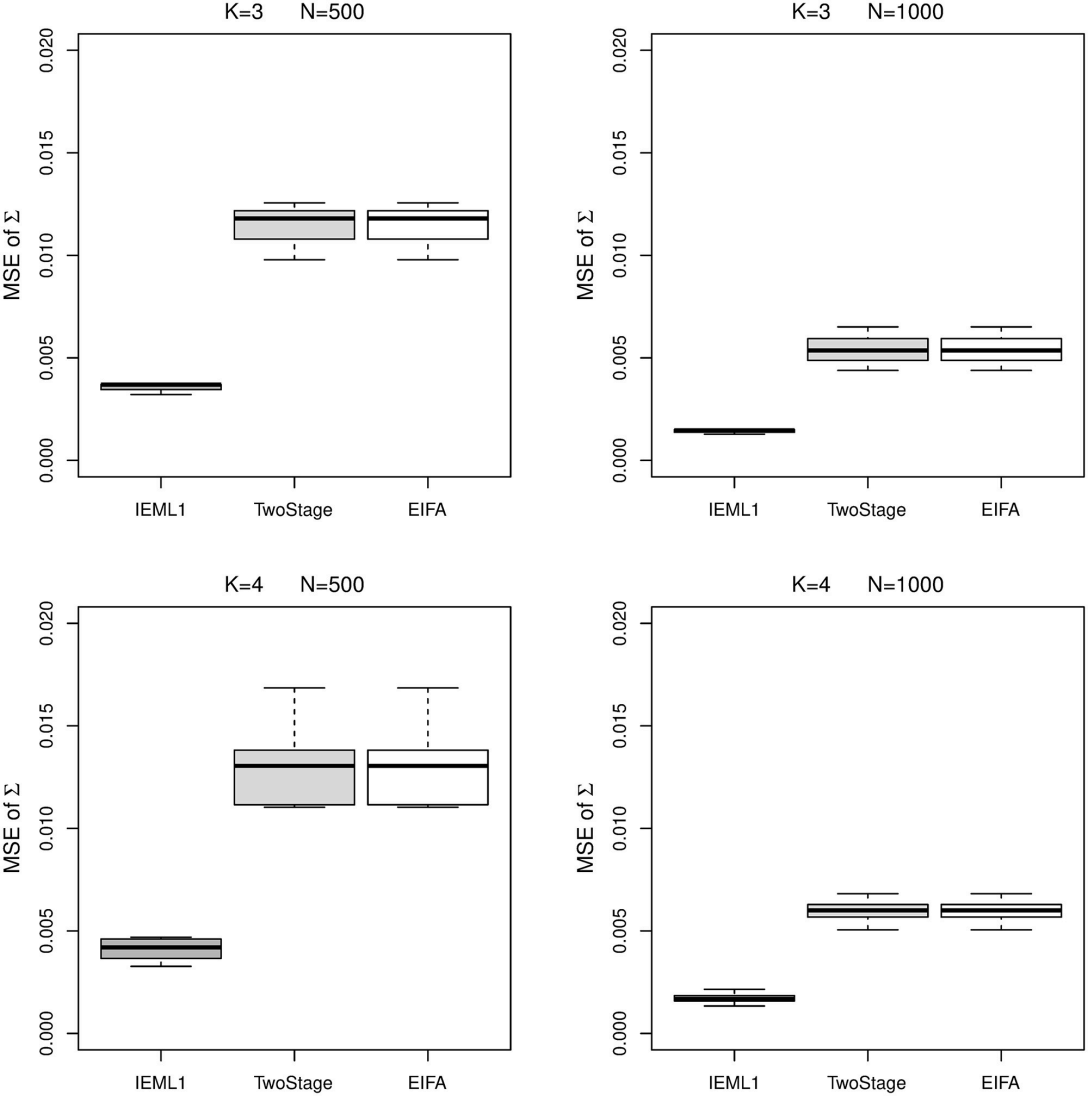
Boxplots of the MSE of Σ obtained by IEML1 (dark gray boxes), two-stage (light gray boxes), EIFAthr and EIFAopt (white boxes) for *K* = 3 and 4 under sample size *N* = 500 and 1000.

Figs 5 and 6 show boxplots of the MSE of ***b*** and Σ obtained by all methods. Note that, EIFAthr and EIFAopt obtain the same estimates of ***b*** and Σ, and consequently, they produce the same MSE of ***b*** and Σ. Therefore, their boxplots of ***b*** and Σ are the same and they are represented by “EIFA” in Figs 5 and 6. We can see that all methods obtain very similar estimates of ***b***. IEML1 gives significant better estimates of Σ than other methods.

### 4.3 Evaluation on heuristic approach for choosing grid points

As presented in the motivating example in Section 3.3, most of the grid points with larger weights are distributed in the cube [−2.4, 2.4]^3^. Intuitively, the grid points for each latent trait dimension can be drawn from the interval [−2.4, 2.4]. In this subsection, we generate three grid point sets denoted by Grid11, Grid7 and Grid5 and compare the performance of IEML1 based on these three grid point sets via simulation study. Specifically, Grid11, Grid7 and Grid5 are three *K*-ary Cartesian power, where 11, 7 and 5 equally spaced grid points on the intervals [−4, 4], [−2.4, 2.4] and [−2.4, 2.4] in each latent trait dimension, respectively.

**Fig 7:**
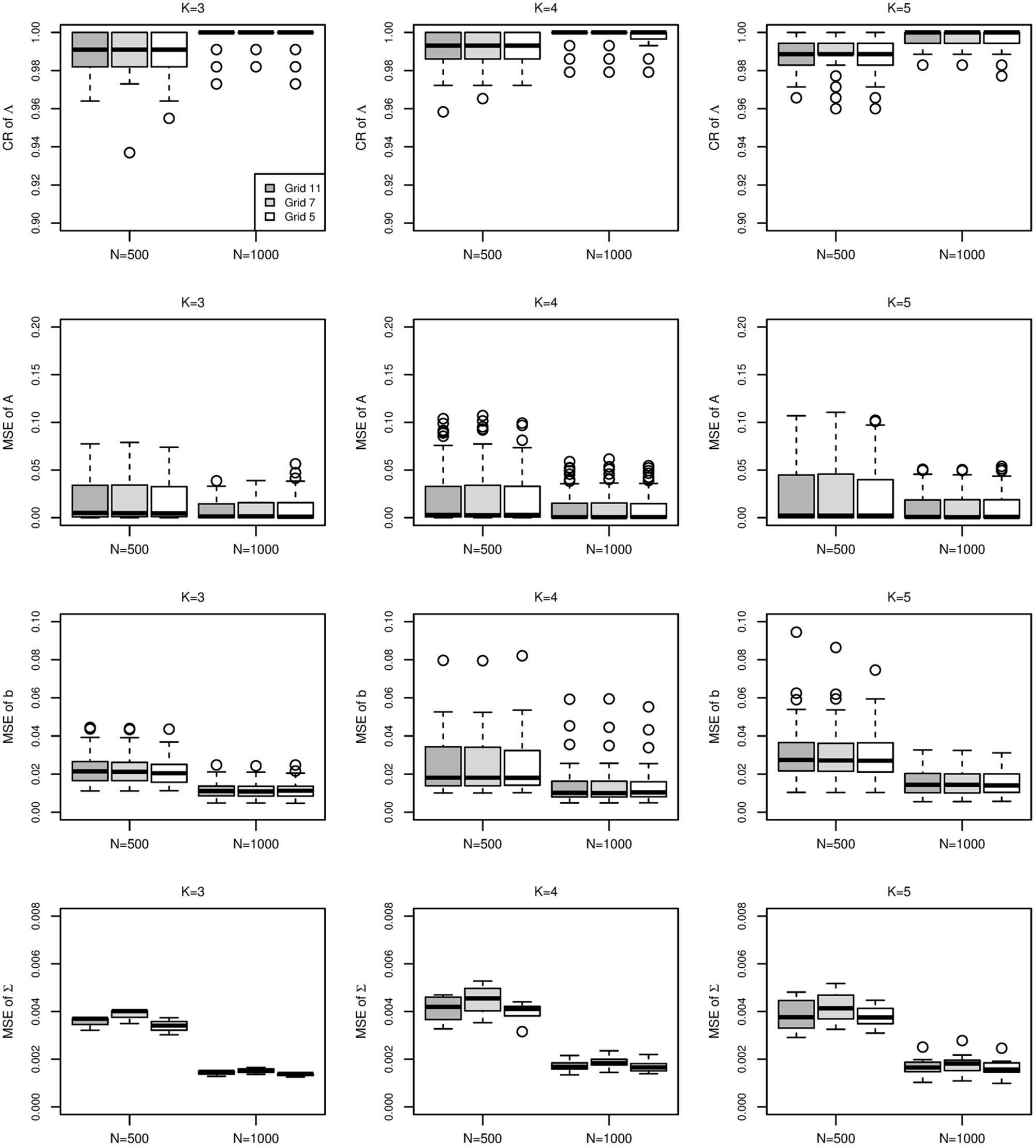
Boxplots of the correct rate of Λ (row 1), the MSE of *A* (row 2), the MSE of *b* (row 3) and the MSE of Σ (row 4) for *K* = 3 (column 1), 4 (column 2) and 5 (column 3) under sample size *N* = 500 and 1000. The dark gray boxes, light gray boxes and white boxes represent the results via 11, 7 and 5 grid points per dimension respectively.

Fig 7 summarizes the boxplots of CRs and MSE of parameter estimates by IEML1 for all cases. From Fig 7, we obtain very similar results when Grid11, Grid7 and Grid5 are used in IEML1. Table 2 shows the average CPU time for all cases. The computing time increases with the sample size and the number of latent traits. The simulation studies show that IEML1 can give quite good results in several minutes if Grid5 is used for M2PL with *K* ≤ 5 latent traits.

**Table 2:** The average CPU time in seconds of IEML1 with Gird11, Grid7 and Grid5 under *K* = 3, 4, 5 with sample size *N* = 500, 1000.

	*K* = 3		*K* = 4		*K* = 5	
	*N* = 500	*N* = 1000	*N* = 500	*N* = 1000	*N* = 500	*N* = 1000
Grid 11	108	134	768	961	9230	14804
Grid 7	68	66	193	287	953	1155
Grid 5	38	53	80	86	209	267

In fact, we also try to use grid point set Grid3 in which each dimension uses three grid points equally spaced in interval [−2.4, 2.4]. But the numerical quadrature with Grid3 is not good enough to approximate the conditional expectation in the E-step. It should be noted that IEML1 may depend on the initial values. In all simulation studies, we use the initial values similarly as described for ***A***_1_ in subsection 4.1. These initial values result in quite good results and they are good enough for practical users in real data applications.

## 5 Real data analysis

In this section, we analyze a data set of the Eysenck Personality Questionnaire given in Eysenck and Barrett [38]. The data set includes 754 Canadian females’ responses (after eliminating subjects with missing data) to 69 dichotomous items, where items 1–25 consist of the psychoticism (P), items 26–46 consist of the extraversion (E) and items 47–69 consist of the neuroticism (N). This data set was also analyzed in Xu et al. [26]. In order to guarantee the psychometric properties of the items, we select those items whose corrected item-total correlation values are greater than 0.2 [39]. The selected items and their original indices are listed in Table 3, with 10, 19 and 23 items corresponding to P, E and N respectively. Items marked by asterisk correspond to negatively worded items whose original scores have been reversed.

**Table 3:** The eysenck pensonality questionnaire items.

1*	P6	Would being in debt worry you?
2	P22	Would you take drugs which may have strange or dangerous effects?
3	P33	Do you enjoy practical jokes that can sometimes really hurt people?
4*	P37	Do good manners and cleanliness matter much to you?
5	P46	Do people who drive carefully annoy you?
6	P50	Do most things taste the same to you?
7*	P57	Do you like to arrive at appointments in plenty of time?
8	P67	Do you think people spend too much time safeguarding their future with savings and insurances?
9	P74	When you catch a train do you often arrive at the last minute?
10	P83	Would you like other people to be afraid of you?
11	E5	Are you a talkative person?
12	E10	Are you rather lively?
13	E14	Can you usually let yourself go and enjoy yourself at a lively party?
14	E17	Do you enjoy meeting new people?
15*	E21	Do you tend to keep in the background on social occasions?
16	E25	Do you like going out a lot?
17*	E29	Do you prefer reading to meeting people?
18	E32	Do you have many friends?
19	E36	Would you call yourself happy-go-lucky?
20	E40	Do you usually take the initiative in making new friends?
21*	E42	Are you mostly quiet when you are with other people?
22	E45	Can you easily get some life into a rather dull party?
23	E49	Do you like telling jokes and funny stories to your friends?
24	E52	Do you like mixing with people?
25	E60	Do you like doing things in which you have to act quickly?
26	E64	Do you often take on more activities than you have time for?
27	E70	Can you get a party going?
28	E82	Do you like plenty of bustle and excitement around you?
29	E86	Do other people think of you as being very lively?
30	N3	Does your mood often go up and down?
31	N7	Do you ever feel “just miserable” for no reason?
32	N12	Do you often worry about things you should not have done or said?
33	N15	Are you an irritable person?
34	N19	Are your feelings easily hurt?
35	N23	Do you often feel “fed-up”?
36	N27	Are you often troubled about feelings of guilt?
37	N31	Would you call yourself a nervous person?
38	N34	Are you a worrier?
39	N38	Do you worry about awful things that might happen?
40	N41	Would you call yourself tense or “highly-strung”?
41	N47	Do you worry about your health?
42	N54	Do you suffer from sleeplessness?
43	N58	Have you often felt listless and tired for no reason?
44	N62	Do you often feel life is very dull?
45	N66	Do you worry a lot about your looks?
46	N68	Have you ever wished that you were dead?
47	N72	Do you worry too long after an embarrassing experience?
48	N75	Do you suffer from “nerves”?
49	N77	Do you often feel lonely?
50	N80	Are you easily hurt when people find fault with you or the work you do?
51	N84	Are you sometimes bubbling over with energy and sometimes very sluggish?
52	N88	Are you touchy about some things?

In the analysis, we designate two items related to each factor for identifiability. Based on the meaning of the items and previous research, we specify items 1 and 9 to P, items 14 and 15 to E, items 32 and 34 to N. We employ the IEML1 to estimate the loading structure and then compute the observed BIC under each candidate tuning parameters in (0.040, 0.038, 0.036, …, 0.002) × *N*, where *N* denotes the sample size 754. The minimal BIC value is 38902.46 corresponding to *η* = 0.02 × *N*. The parameter estimates of ***A*** and ***b*** are given in Table 4, and the estimate of Σ is

$$\hat{\Sigma }=(\begin{array}{ccc} 1.000 & 0.121 & -0.018\\ 0.121 & 1.000 & -0.246\\ -0.018 & -0.246 & 1.000 \end{array}).$$

**Table 4:** The parameter estimates by the IEML1 algorithm for the real data.

	*A*			*b*		*A*			*b*
1	0.723	0.000	0.000	-1.943	27	0.000	1.839	0.000	0.745
2	0.659	0.000	0.000	-2.181	28	0.000	1.311	0.000	1.479
3	1.097	0.000	0.000	-3.510	29	0.000	1.699	0.000	1.469
4	0.966	0.000	0.000	-3.175	30	0.321	0.000	1.167	0.769
5	1.177	0.000	0.000	-1.779	31	0.000	0.000	0.890	0.911
6	0.854	0.000	0.000	-3.093	32	0.000	0.000	1.321	2.082
7	1.689	0.000	0.000	-1.952	33	0.000	0.000	0.887	-1.173
8	0.515	0.000	0.000	-1.079	34	0.000	0.000	1.356	1.264
9	2.030	0.000	0.000	-1.718	35	0.000	-0.153	1.430	0.399
10	1.075	0.000	0.000	-3.316	36	0.000	0.000	1.473	0.393
11	0.000	1.542	0.000	1.128	37	0.000	0.000	1.228	-0.562
12	0.000	1.683	0.000	2.283	38	-0.392	0.000	1.679	1.284
13	0.000	1.397	0.000	1.931	39	0.000	0.000	1.197	0.350
14	0.000	1.324	0.000	2.872	40	0.422	0.000	1.475	-1.440
15	0.000	1.696	0.000	0.702	41	-0.248	0.188	0.751	0.311
16	0.336	0.840	0.000	1.294	42	0.255	0.000	0.784	-0.408
17	0.000	1.143	0.000	2.073	43	0.000	0.000	1.168	0.777
18	0.000	1.200	0.000	2.075	44	0.000	-0.247	1.023	-1.214
19	0.000	0.787	-0.463	-0.133	45	0.000	0.225	1.105	0.587
20	0.000	1.617	0.000	0.716	46	0.000	-0.128	0.663	0.057
21	0.000	1.831	0.000	0.983	47	-0.380	-0.253	1.568	0.898
22	0.000	1.962	0.000	-0.066	48	0.000	0.000	1.749	-0.878
23	0.000	0.970	0.000	2.025	49	0.000	-0.220	1.199	-0.327
24	0.000	2.180	0.000	3.455	50	0.000	-0.188	1.126	0.997
25	0.358	0.631	-0.232	0.541	51	0.000	0.000	0.912	1.889
26	0.245	0.478	0.000	0.584	52	0.000	0.000	0.829	2.085

From the results, most items are found to remain associated with only one single trait while some items related to more than one trait. Most of these findings are sensible. For example, item 19 (‘Would you call yourself happy-go-lucky?’) designed for extraversion is also related to neuroticism which reflects individuals’ emotional stability. Item 49 (‘Do you often feel lonely?’) is also related to extraversion whose characteristics are enjoying going out and socializing. In addition, it is reasonable that item 30 (‘Does your mood often go up and down?’) and item 40 (‘Would you call yourself tense or ‘highly-strung’?’) are related to both neuroticism and psychoticism.

## 6 Concluding remarks

In this paper, we obtain a new weighted log-likelihood based on a new artificial data set for M2PL models, and consequently we propose IEML1 to optimize the *L*_1_-penalized log-likelihood for latent variable selection. We give a heuristic approach for choosing the quadrature points used in numerical quadrature in the E-step, which reduces the computational burden of IEML1 significantly. There are three advantages of IEML1 over EML1, the two-stage method, EIFAthr and EIFAopt. First, the computational complexity of M-step in IEML1 is reduced to *O*(2 × *G*) from *O*(*N* × *G*). In our simulation studies, IEML1 needs a few minutes for M2PL models with no more than five latent traits. Second, IEML1 updates covariance matrix Σ of latent traits and gives a more accurate estimate of Σ. Third, IEML1 outperforms the two-stage method, EIFAthr and EIFAopt in terms of CR of the latent variable selection and the MSE for the parameter estimates.

The current study will be extended in the following directions for future research. First, we will generalize IEML1 to multidimensional three-parameter (or four parameter) logistic models that give much attention in recent years. Second, other numerical integration such as Gaussian-Hermite quadrature [4, 29] and adaptive Gaussian-Hermite quadrature [34] can be adopted in the E-step of IEML1. Gaussian-Hermite quadrature uses the same fixed grid point set for each individual and can be easily adopted in the framework of IEML1. However, further simulation results are needed. Compared to the Gaussian-Hermite quadrature, the adaptive Gaussian-Hermite quadrature produces an accurate fast converging solution with as few as two points per dimension for estimation of MIRT models [34]. Therefore, the adaptive Gaussian-Hermite quadrature is also potential to be used in penalized likelihood estimation for MIRT models although it is impossible to get our new weighted log-likelihood in Eq (15) due to applying different grid point set for different individual. Third, we will accelerate IEML1 by parallel computing technique for medium-to-large scale variable selection, as [40] produced larger gains in performance for MIRT estimation by applying the parallel computing technique. Fourth, the new weighted log-likelihood on the new artificial data proposed in this paper will be applied to the EMS in [26] to reduce the computational complexity for the MS-step.

## Supporting information

S1 Appendix True discrimination and difficulty parameters in simulation studies. (PDF)

S2 Appendix FNR, FPR and precision of the loading structure in the simulation for the unknown Σ case. (PDF)

S3 Appendix Data sets of the study. (PDF)

S4 Appendix R codes of IEML1. (PDF)
