# Supplementary material for: Accelerating L1-penalized expectation maximization algorithm for latent variable selection in multidimensional two-parameter logistic models
Source: PLoS One. 2023 Jan 17;18(1):e0279918. doi: 10.1371/journal.pone.0279918 (PMC9844851; doi:10.1371/journal.pone.0279918)
Supplement: S1 Appendix — (PDF) [file pone.0279918.s001.pdf]

S1 Appendix

True discrimination and difficulty parameters in simulation studies.

Table A. The true discrimination matrix $\boldsymbol{A}_1$ with $K = 3$ and $J = 40$.

	**Items**									
**Latent traits**	**1**	**2**	**3**	**4**	**5**	**6**	**7**	**8**	**9**	**10**
1	1.763	1.583	1.298	1.296	1.036	0.828	0.749	0.745	0.557	0.000
2	0.000	0.000	0.000	0.000	0.000	0.000	0.000	0.000	0.000	1.863
3	0.000	0.000	0.000	0.000	0.000	0.000	0.000	0.000	0.000	0.000
	**Items**									
**Latent traits**	**11**	**12**	**13**	**14**	**15**	**16**	**17**	**18**	**19**	**20**
1	0.000	0.000	0.000	0.000	0.000	0.000	0.000	0.000	0.000	0.000
2	1.808	1.690	1.540	1.422	1.369	1.088	0.994	0.524	0.000	0.000
3	0.000	0.000	0.000	0.000	0.000	0.000	0.000	0.000	1.988	1.922
	**Items**									
**Latent traits**	**21**	**22**	**23**	**24**	**25**	**26**	**27**	**28**	**29**	**30**
1	0.000	0.000	0.000	0.000	0.000	0.000	0.000	1.187	1.005	0.722
2	0.000	0.000	0.000	0.000	0.000	0.000	0.000	1.407	1.276	1.213
3	1.808	1.746	1.702	1.265	1.029	1.009	0.619	0.000	0.000	0.000
	**Items**									
**Latent traits**	**31**	**32**	**33**	**34**	**35**	**36**	**37**	**38**	**39**	**40**
1	1.808	1.310	0.954	0.000	0.000	0.000	0.702	1.072	1.189	0.694
2	0.000	0.000	0.000	1.759	1.070	1.735	1.171	0.951	0.543	0.920
3	1.574	1.669	1.407	1.903	1.775	0.757	1.030	0.823	0.576	0.567

Table B. The true difficulty parameters $\boldsymbol{b}_1$.

**Items**	**Difficulty parameters**									
1-10	1.216	1.354	0.734	0.863	-0.925	-0.768	2.159	-1.326	-0.411	-0.565
11-20	-1.005	-0.203	-0.663	-1.317	-0.611	1.596	-0.086	-1.723	1.505	-0.686
21-30	0.436	1.125	1.084	0.436	-0.156	-0.203	1.071	-1.605	-0.904	-1.233
31-40	-0.453	-0.039	-0.728	1.480	1.133	-0.346	-0.472	0.678	-1.120	-1.792

Table C. The true discrimination matrix $\boldsymbol{A}_2$ with $K = 4$ and $J = 40$.

	Items									
**Latent traits**	**1**	**2**	**3**	**4**	**5**	**6**	**7**	**8**	**9**	**10**
1	1.669	1.374	1.356	1.356	1.095	0.901	0.000	0.000	0.000	0.000
2	0.000	0.000	0.000	0.000	0.000	0.000	1.689	1.620	1.298	1.026
3	0.000	0.000	0.000	0.000	0.000	0.000	0.000	0.000	0.000	0.000
4	0.000	0.000	0.000	0.000	0.000	0.000	0.000	0.000	0.000	0.000
	**Items**									
**Latent traits**	**11**	**12**	**13**	**14**	**15**	**16**	**17**	**18**	**19**	**20**
1	0.000	0.000	0.000	0.000	0.000	0.000	0.000	0.000	0.000	0.000
2	0.824	0.763	0.000	0.000	0.000	0.000	0.000	0.000	0.000	0.000
3	0.000	0.000	1.986	1.800	1.627	0.998	0.906	0.649	0.000	0.000
4	0.000	0.000	0.000	0.000	0.000	0.000	0.000	0.000	1.687	1.531
	**Items**									
**Latent traits**	**21**	**22**	**23**	**24**	**25**	**26**	**27**	**28**	**29**	**30**
1	0.000	0.000	0.000	0.000	1.062	0.795	1.477	0.821	1.754	0.503
2	0.000	0.000	0.000	0.000	1.129	1.007	0.000	0.000	0.000	0.000
3	0.000	0.000	0.000	0.000	0.000	0.000	0.886	1.195	0.000	0.000
4	1.095	1.094	0.821	0.781	0.000	0.000	0.000	0.000	1.650	0.547
	**Items**									
**Latent traits**	**31**	**32**	**33**	**34**	**35**	**36**	**37**	**38**	**39**	**40**
1	0.000	0.000	0.000	0.000	0.000	0.000	1.453	1.402	1.629	0.000
2	1.702	1.097	0.658	1.322	0.000	0.000	1.228	1.926	0.000	1.540
3	1.672	1.588	0.000	0.000	1.851	0.574	1.737	0.000	1.816	1.351
4	0.000	0.000	1.354	0.613	0.994	1.006	0.000	1.478	1.804	1.473

Table D. The true difficulty parameters $\boldsymbol{b}_2$.

**Items**	**Difficulty parameters**									
1-10	-0.267	0.394	0.182	-0.448	-0.443	0.182	-0.478	1.409	0.673	-2.035
11-20	-0.528	-0.640	1.243	-0.142	-0.404	0.312	1.194	-1.962	-1.070	-2.036
21-30	-0.386	0.559	0.858	-1.750	-1.940	0.062	-0.252	-1.470	0.397	-0.809
31-40	0.202	-0.859	-0.004	0.260	0.860	0.467	2.496	1.160	1.662	-1.177

Table E. The true discrimination matrix $\boldsymbol{A}_3$ with $K = 5$ and $J = 40$.

	**Items**									
**Latent traits**	**1**	**2**	**3**	**4**	**5**	**6**	**7**	**8**	**9**	**10**
1	1.970	1.871	1.826	0.558	0.000	0.000	0.000	0.000	0.000	0.000
2	0.000	0.000	0.000	0.000	1.701	1.657	1.563	1.433	0.000	0.000
3	0.000	0.000	0.000	0.000	0.000	0.000	0.000	0.000	1.937	1.722
4	0.000	0.000	0.000	0.000	0.000	0.000	0.000	0.000	0.000	0.000
5	0.000	0.000	0.000	0.000	0.000	0.000	0.000	0.000	0.000	0.000
	**Items**									
**Latent traits**	**11**	**12**	**13**	**14**	**15**	**16**	**17**	**18**	**19**	**20**
1	0.000	0.000	0.000	0.000	0.000	0.000	0.000	0.000	0.000	0.000
2	0.000	0.000	0.000	0.000	0.000	0.000	0.000	0.000	0.000	0.000
3	1.354	1.304	0.000	0.000	0.000	0.000	0.000	0.000	0.000	0.000
4	0.000	0.000	1.910	1.712	0.984	0.593	0.000	0.000	0.000	0.000
5	0.000	0.000	0.000	0.000	0.000	0.000	1.844	1.827	1.670	0.954
	**Items**									
**Latent traits**	**21**	**22**	**23**	**24**	**25**	**26**	**27**	**28**	**29**	**30**
1	1.968	1.351	1.429	0.825	0.000	0.000	0.000	0.000	0.000	0.000
2	0.904	0.000	0.000	0.000	1.731	1.454	1.790	0.000	0.000	0.000
3	0.000	1.696	0.000	0.000	1.882	0.000	0.000	1.989	1.018	0.000
4	0.000	0.000	1.671	0.000	0.000	0.819	0.000	1.631	0.000	1.200
5	0.000	0.000	0.000	1.401	0.000	0.000	1.612	0.000	1.807	0.772
	**Items**									
**Latent traits**	**31**	**32**	**33**	**34**	**35**	**36**	**37**	**38**	**39**	**40**
1	1.385	1.720	1.731	0.652	1.434	1.472	0.000	0.000	0.000	0.000
2	1.691	1.182	1.562	0.000	0.000	0.000	1.348	1.694	1.850	0.000
3	1.079	0.000	0.000	1.966	0.705	0.000	1.498	1.398	0.000	0.914
4	0.000	1.513	0.000	1.491	0.000	1.123	0.530	0.000	1.949	1.411
5	0.000	0.000	1.977	0.000	0.957	0.876	0.000	1.256	1.251	0.525

Table F. The true difficulty parameters $\boldsymbol{b}_3$.

**Items**	**Difficulty parameters**									
1-10	1.484	-0.262	-0.793	0.811	0.467	-1.087	0.201	1.655	0.635	-1.050
11-20	0.508	0.979	1.110	0.524	-2.199	-0.733	1.955	0.219	-1.552	0.926
21-30	0.596	-0.375	1.188	-0.479	-1.036	0.746	-0.708	-1.803	-0.462	-0.899
31-40	-1.079	-1.032	0.594	0.531	0.967	0.006	0.721	-1.134	0.958	-0.459
