# Supplementary material for: Accelerating L1-penalized expectation maximization algorithm for latent variable selection in multidimensional two-parameter logistic models
Source: PLoS One. 2023 Jan 17;18(1):e0279918. doi: 10.1371/journal.pone.0279918 (PMC9844851; doi:10.1371/journal.pone.0279918)
Supplement: S2 Appendix — (PDF) [file pone.0279918.s002.pdf]

S2 Appendix

FNR, FPR and precision of the loading structure in the simulation for the unknown Σ case.

As complements to the correct rate (CR) in subsection 4.2 (i.e., simulation for the unknown Σ case), the false negative rate (FNR), false positive rate (FPR) and precision are used to compare the latent variable selection performance of the IEML1, two-stage method, EIFAthr and EIFAopt. These metrics are calculated as follows,

$$\text{FNR} = \frac{\sum_{j=1}^{J}\sum_{k=1}^{K}\lambda_{jk}(1-\hat{\lambda}_{jk})}{\sum_{j=1}^{J}\sum_{k=1}^{K}\lambda_{jk}}, \quad \text{FPR} = \frac{\sum_{j=1}^{J}\sum_{k=1}^{K}(1-\lambda_{jk})\hat{\lambda}_{jk}}{\sum_{j=1}^{J}\sum_{k=1}^{K}(1-\lambda_{jk})}, \quad \text{precision} = \frac{\sum_{j=1}^{J}\sum_{k=1}^{K}\lambda_{jk}\hat{\lambda}_{jk}}{\sum_{j=1}^{J}\sum_{k=1}^{K}\hat{\lambda}_{jk}},$$

where λ_{jk} is the (j,k)-element in the true loading structure Λ and $\hat{\lambda}_{jk}$ is the estimate of λ_{jk}.

The boxplots of FNR, FPR and precision over 100 replications for $K = 3, 4$ under sample size $N = 500, 1000$ are summarized in Fig A, Fig B and Fig C, respectively. From Fig A we can see that IEML1 performs the best in FNR. IEML1 also has good performance in FPR and precision from Fig B and Fig C. Although EIFAthr achieves the highest precision and lowest FPR when the threshold is larger than 0.55, it performs the worst in terms of FNR. Therefore, the EIFAthr cannot have good performance in all three metrics. EIFAopt and the two-stage method perform very similarly as IEML1 in FNR, but they are not better than IEML1 in FPR and precision. Overall, IEML1 performs very well in all three metrics.

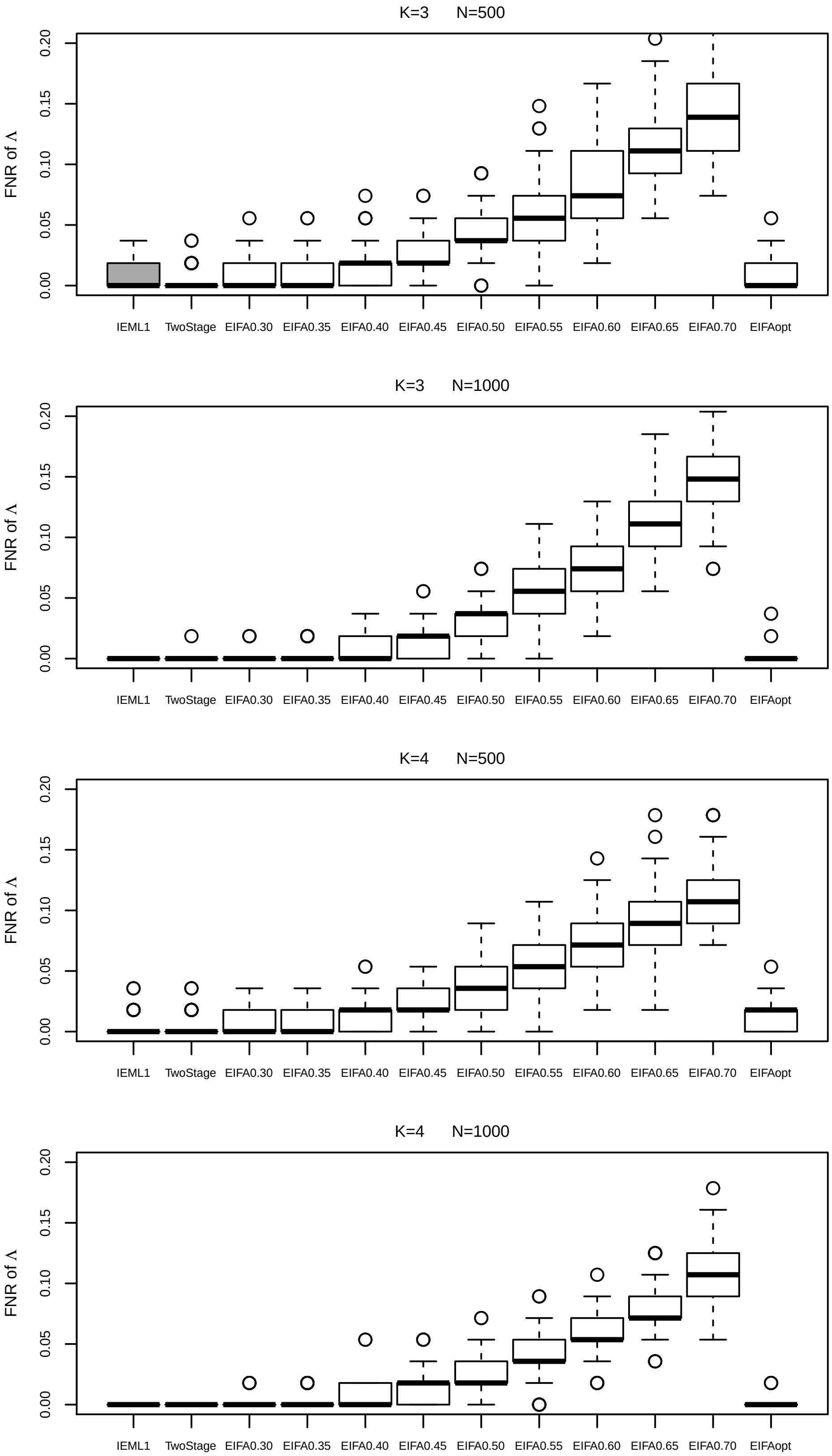

Fig A. Boxplots of the false negative rate (FNR) of Λ obtained by IEML1 (dark gray boxes), two-stage (light gray boxes), EIFAthr and EIFAopt (white boxes) for $K = 3, 4$ under sample size $N = 500, 1000$.

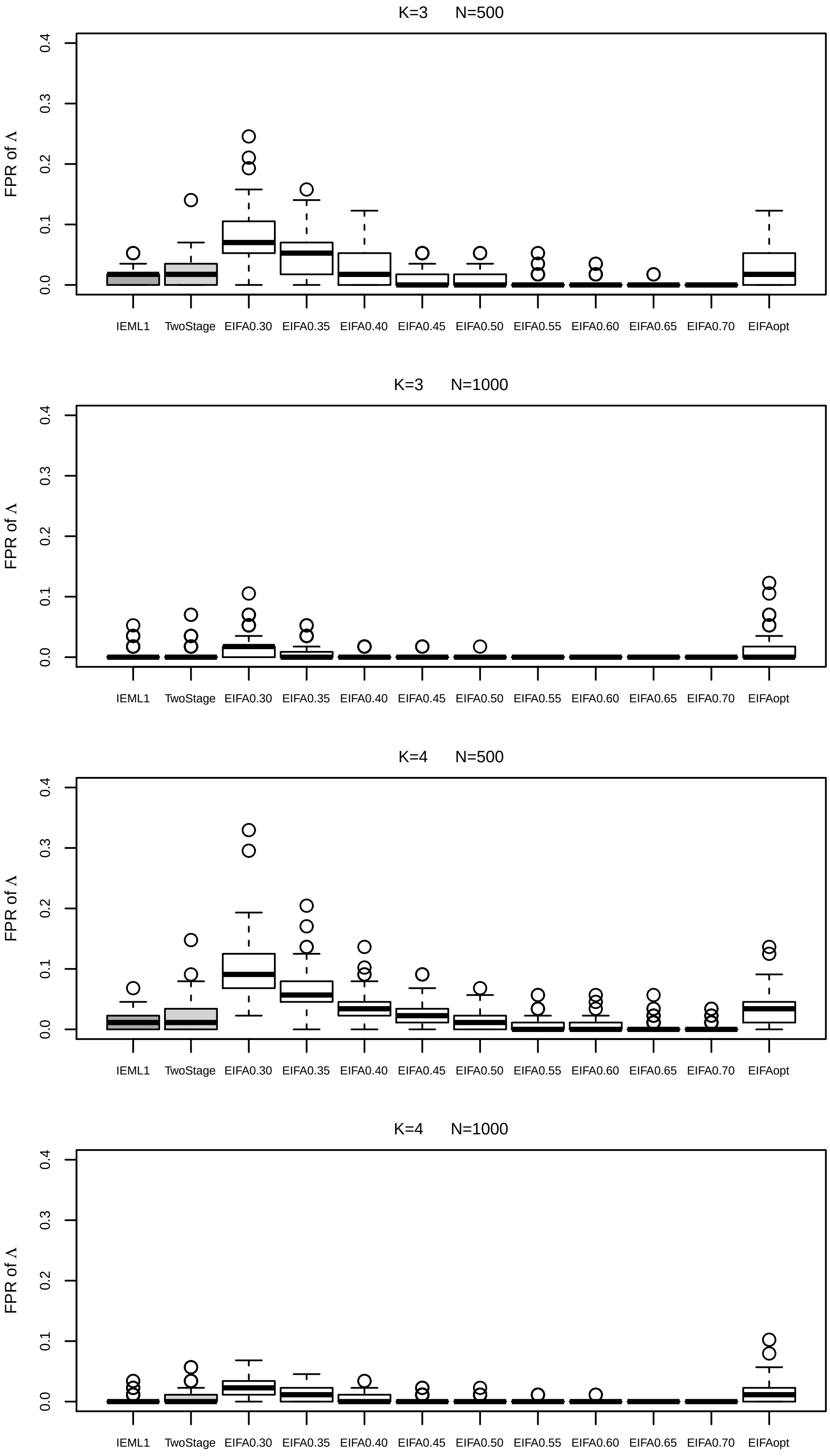

Fig B. Boxplots of the false positive rate (FPR) of Λ obtained by IEML1 (dark gray boxes), two-stage (light gray boxes), EIFAthr and EIFAopt (white boxes) for $K = 3, 4$ under sample size $N = 500, 1000$.

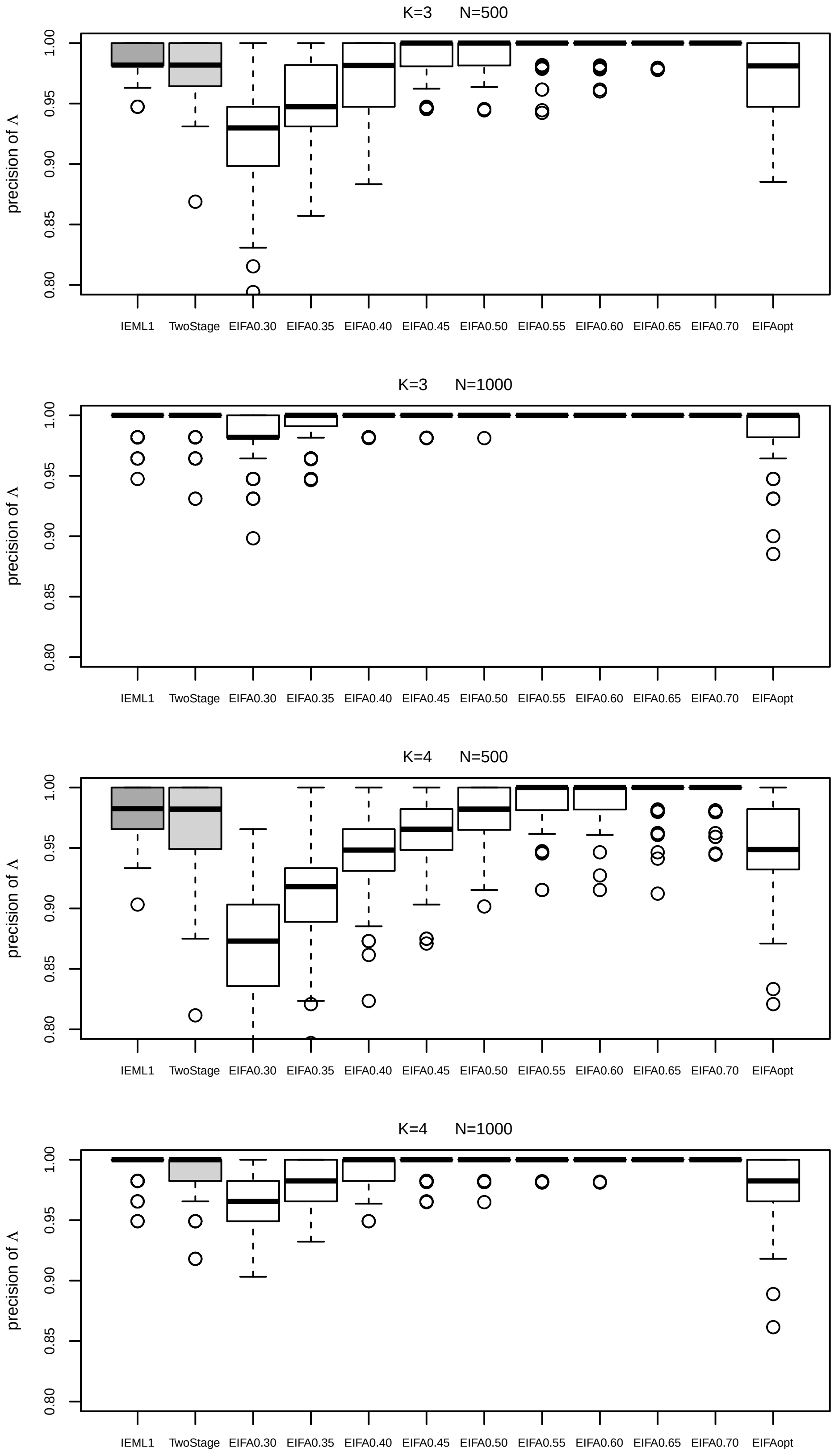

Fig C. Boxplots of the precision of Λ obtained by IEML1 (dark gray boxes), two-stage (light gray boxes), EIFAthr and EIFAopt (white boxes) for $K = 3, 4$ under sample size $N = 500, 1000$.
