# Supplementary material for: Accelerating L1-penalized expectation maximization algorithm for latent variable selection in multidimensional two-parameter logistic models
Source: PLoS One. 2023 Jan 17;18(1):e0279918. doi: 10.1371/journal.pone.0279918 (PMC9844851; doi:10.1371/journal.pone.0279918)
Supplement: S3 Appendix — (PDF) [file pone.0279918.s003.pdf]

S3 Appendix

Data sets of the study.

In this appendix, we provide the source of the public data set of Eysenck Personality Questionnaire. Moreover, our R codes that are used for the generation of the response data in the simulation studies are shared so that interested readers can replicate the simulation results.

1. Data set of Eysenck Personality Questionnaire used in Section 5

The data set of the Eysenck Personality Questionnaire used in Section 5 (i.e., Real data analysis) is publicly available in the supplementary data of Eysenck and Barrett (2013). This data set can be directly downloaded from https://www.sciencedirect.com/science/article/pii/S0191886912004825.

2. R codes for generating the response data sets in the simulation studies

```
library(magrittr)
library(MASS)

# ---- data generator function -------------------------------------------------
DataGen <- function(A_t, b_t, sigma_t, N, seed_num){

  set.seed(seed_num)

  J <- ncol(A_t)
  K <- nrow(A_t)

  theta <- mvrnorm(n=N, mu=rep(0,K), Sigma=sigma_t) # latent traits
  y <- theta %>%
    `%*%` (A_t) %>%
    `+` (matrix(data=b_t,nrow=N,ncol=J,byrow=T)) %>%
    plogis(q=.) %>%
    rbinom(n=N*J, size=1, prob=.) %>%
    matrix(data=., nrow=N, ncol=J, byrow=F)

  storage.mode(y) <- "integer"
  return(list(y=y,theta=theta))
}
# ------------------------------------------------------------------------------

# ---- K = 3, 4, 5 ----
# ---- J = 40 ----
# ---- N = 500, 1000 ----
# ---- off-diagonal of sigma is 0.1 ----
setwd("../Rcode_for_upload")
load(file="./TrueParam_K3J40.Rdata") # load true A & b

J <- ncol(A_t)  # NO. of items
K <- nrow(A_t)  # NO. of latent traits

M <- 100       # NO. of data set
N <- 1000      # NO. of subjects for each data set
s <- 0.1       # off-diagonal elements of the true sigma
```

```
Sigma_t <- matrix(s,K,K); diag(Sigma_t) <- 1

# ---- generate random samples ----
set.seed(N+K*10+s*10)
seed_vec <- sample(1e6:(1e7-1), M, replace=FALSE)


for(m in 1:M){

  seed_num <- seed_vec[m]

  output <- DataGen(A_t, b_t, Sigma_t, N, seed_num)
  y      <- output$y
  theta  <- output$theta

  colnames(theta) <- paste("trait", 1:K, sep="")
  colnames(y)     <- paste("item", sprintf("%02d", 1:J), sep="")

  # ---- save data set ----
  save(seed_num, A_t, b_t, Sigma_t, y, theta,
       file=sprintf("./K%dJ%dS%02dN%d/dataset%03d.Rdata",
                    K,J,as.integer(s*10),N,m)
       )

}

# ---- display y[1:5,1:10] ----
load(file="./K3J40S01N1000/dataset001.Rdata")

print(y[1:5,1:10])

#      item01 item02 item03 item04 item05 item06 item07 item08 item09 item10
# [1,]      0      1      1      1      0      0      0      0      1      0
# [2,]      1      1      0      1      1      0      1      0      0      1
# [3,]      1      1      1      1      1      1      1      1      0      0
# [4,]      1      0      0      0      1      0      0      0      0      0
# [5,]      1      0      1      1      0      1      1      1      0      1
```
