# Supplementary material for: Accelerating L1-penalized expectation maximization algorithm for latent variable selection in multidimensional two-parameter logistic models
Source: PLoS One. 2023 Jan 17;18(1):e0279918. doi: 10.1371/journal.pone.0279918 (PMC9844851; doi:10.1371/journal.pone.0279918)
Supplement: S4 Appendix — (PDF) [file pone.0279918.s004.pdf]

S4 Appendix

R codes of IEML1.

The R codes of the IEML1 method are provided as follows.

1. R codes of the IEML1 method

```
library(glmnet)
library(progress)
Rcpp::sourceCpp("./IEML1_calcu.cpp")

# ---- some useful function ----
grid_pts <- function(K, lb=-4, ub=4, np=11){
  # K     : No. of latent variables.
  # lb, ub: lower bound & upper bound.
  # np    : No. of grid points for each dimension.
  # output: grid points.

  p_list <- list()
  for(k in 1:K){
    p_list[[k]] <- seq(from=lb, to=ub, length.out=np)
  }
  grid_pts <- as.matrix(expand.grid(p_list, stringsAsFactors=FALSE))
  colnames(grid_pts) <- NULL
  return(grid_pts)
}

calcu_sigma_hat <- function(x_grid, w0, N){
  sigma_hat <- t(x_grid)%*%(x_grid*w0)/N
  sigma_hat <- 0.5*sigma_hat + 0.5*t(sigma_hat)
}

calcu_q0 <- function(S, sigma, N){
  - N/2*log(det(sigma)) - N/2*sum(solve(sigma)*S)
}


# ---- IEML1 for M2pl ----
M2pl_IEML1 <- function(
  y,              # responses of all subjects     (N*J mat)
  A_init,         # initial value of A            (K*J mat)
  b_init,         # initial value of b            (1*J vec)
  Sigma_init,     # initial value of Sigma        (K*K mat)
  fixed,          # the items whose sub-models are fixed
  eta,            # penalized parameter with positive value
  grid_num = 5,   # No. of grid points per dimension
  lb = -4, ub = 4,
  Max_iter = 50,  # maximal iteration number
  is.SigmaKnown = FALSE)
{

  J <- ncol(A_init)  # number of items
  K <- nrow(A_init)  # number of latent trait
  N <- nrow(y)      # number of subjects
```

```
# ---- grid points ----
x_grid <- grid_pts(K=K, lb=lb, ub=ub, np=grid_num)
G      <- nrow(x_grid)

# ---- augmented data in M-step for glmnet ----
y_aug <- c(rep(1,G), rep(0,G))
x_aug <- rbind(x_grid, x_grid)

# ---- Parameter Initialization ----
A_c     <- A_init;       A_new     <- A_c
b_c     <- b_init;       b_new     <- b_c
Sigma_c <- Sigma_init;    Sigma_new <- Sigma_c

Mod_c   <- A_c!=0;       Mod_new   <- Mod_c
Q_c     <- -Inf;         Q_new     <- Q_c

Q0 <- 0
Qj_vec <- rep(0,J)

# ---- IEML1 iteration ----
time_IEML1  <- proc.time()
iter_IEML1  <- 0

while(iter_IEML1 < Max_iter){

  iter_IEML1 <- iter_IEML1 + 1

  pb_em <- progress_bar$new(
            format=sprintf("IEML1:%03d [:bar] :percent eta::eta", iter_IEML1),
            total=J+2, clear=TRUE, width=60, show_after=0
           )
  pb_em$tick(0)  # progress bar

  # ---- E-step: ----
  Estep_output <- EstepSpd(y=y, x=x_grid, A=A_c, b=b_c, sigma=Sigma_c)
  w0 <- Estep_output$w0
  w  <- Estep_output$w
  pb_em$tick(1)

  # ---- M-step: ----
  # ---- estimate correlation matrix -----
  if(is.SigmaKnown){
    Sigma_new <- Sigma_c
    Q0 <- 0
  }
  else{
    Sigma_hat <- calcu_sigma_hat(x_grid, w0, N)
    Sigma_new <- calcu_sigma_cmle_cpp(Sigma_hat, Sigma_c, tol=1e-4)
    Q0 <- calcu_q0(S=Sigma_hat, sigma=Sigma_new, N)
  }

  pb_em$tick(1)

  # ---- estimate item parameters -----
  for(j in fixed){

    excl <- which(A_init[,j]==0)
```

```
    fit <- glmnet(x=x_aug, y=y_aug, weights=w[,j], family="binomial",
                  alpha=1, lambda=eta, standardize=FALSE, exclude=excl)

    A_new[,j]  <- as.vector(coef(fit)[-1])
    b_new[j]   <- as.numeric(coef(fit)[1])
    Qj_vec[j]  <- -1/2*(1-fit$dev.ratio)*fit$nulldev - eta*sum(abs(A_new[,j]))

    pb_em$tick(1)
  }

  for(j in (1:J)[-fixed]){

    fit <- glmnet(x=x_aug, y=y_aug, weights=w[,j], family="binomial",
                  alpha=1, lambda=eta, standardize=FALSE)

    A_new[,j]  <- as.vector(coef(fit)[-1])
    b_new[j]   <- as.numeric(coef(fit)[1])
    Qj_vec[j]  <- -1/2*(1-fit$dev.ratio)*fit$nulldev - eta*sum(abs(A_new[,j]))

    pb_em$tick(1)
  }

  Mod_new <- A_new!=0
  Q_new   <- sum(Qj_vec) + Q0

  # ---- display the new parameters ----
  # cat("A_new:\n");      print(A_new)
  # cat("b_new:\n");      print(b_new)
  # cat("Sigma_new:\n");   print(Sigma_new)
  # cat("Q_new:", Q_new, "\n");

  # ---- Stop criterion ----
  if(all(Mod_new == Mod_c)){
    err_A     <- sqrt(sum((A_new-A_c)^2)/sum(A_c^2))
    err_Sigma <- sqrt(sum((Sigma_new-Sigma_c)^2)/sum(Sigma_c^2))

    if(all(c(err_A, err_Sigma) < 0.01)) {break}
  }

  if(iter_IEML1 > 1){
    if(abs((Q_new-Q_c)/Q_c) < 1e-3) {break}
  }

  # ---- replace current parameter ----
  A_c     <- A_new
  b_c     <- b_new
  Sigma_c <- Sigma_new
  Mod_c   <- Mod_new
  Q_c     <- Q_new

} # end while EML1

time_IEML1 <- proc.time() - time_IEML1
time_IEML1 <- as.numeric(time_IEML1[3])

# ---- Return output ----
result <- list(A = A_new,
               b = b_new,
```

```
                  Sigma = Sigma_new,
                  iter = iter_IEML1,
                  time = time_IEML1
  )
  return(result)
}


# ---- a simple test ----
if(sys.nframe() == 0L){

  library(magrittr)
  library(MASS)

  A_t <- c(2.0,1.5,1.0,0.5,0.0,0.0,0.0,0.0,0.0,0.0,0.0,0.0,
           0.0,0.0,0.0,0.0,2.0,1.5,1.0,0.5,0.0,0.0,0.0,0.0,
           0.0,0.0,0.0,0.0,0.0,0.0,0.0,0.0,2.0,1.5,1.0,0.5)
  A_t <- matrix(A_t, nrow=3, ncol=12, byrow=T)
  fixed <- c(1,5,9)  # for identification

  J <- ncol(A_t)  # No. of items
  K <- nrow(A_t)  # No. of latent traits
  N <- 1000       # No. of subjects

  # ---- true parameter setting ----
  b_t     <- rep(0,J)
  sigma_t <- matrix(.1,K,K); diag(sigma_t) <- 1

  # ---- generate random sample ----
  set.seed(1)
  x <- mvrnorm(n=N, mu=rep(0,K), Sigma=sigma_t) # latent traits
  y <- x %>%
    `%*%` (A_t) %>%
    `+` (matrix(data=b_t,nrow=N,ncol=J,byrow=T)) %>%
    plogis(q=.) %>%
    rbinom(n=N*J, size=1, prob=.) %>%
    matrix(data=., nrow=N, ncol=J, byrow=F)

  A_init <- matrix(data=1/J, nrow=K, ncol=J, byrow=TRUE); A_init[,fixed] <- diag(1,K)
  b_init <- rep(0,J)
  Sigma_init <- diag(1,K)

  grid_num <- 11
  eta      <- .03

  # ---- IEML1 ----
  output <- M2pl_IEML1(y = y,
                       A_init     = A_init,
                       b_init     = b_init,
                       Sigma_init = sigma_t,
                       fixed = fixed,
                       eta   = eta,
                       grid_num = grid_num
  )

  print(output)
}
```

2. The cpp file IEML1_calcu.cpp

```
#define RCPP_ARMADILLO_RETURN_ANYVEC_AS_VECTOR
#include <RcppArmadillo.h>
// [[Rcpp::depends(RcppArmadillo)]]
using namespace Rcpp;

arma::vec log_dmvnorm(arma::mat sigma, arma::mat x){

  int G = x.n_rows;
  arma::mat omega = arma::inv(sigma);
  arma::vec log_phi(G);

  int g;
  arma::mat s(1,1);
  for(g=0; g<G; g++){
    s = - 1.0/2*(x.row(g))*omega*(x.row(g).t());
    log_phi(g) = s(0,0);
  }

  return log_phi;
}


// [[Rcpp::export]]
Rcpp::List EstepSpd(arma::mat y,
                    arma::mat x,
                    arma::mat A,
                    arma::rowvec b,
                    arma::mat sigma
){

  int N = y.n_rows; // number of subjects
  int J = y.n_cols; // number of items
  int G = x.n_rows; // number of grid points

  // -- pre-computation --
  arma::vec log_phi_x = log_dmvnorm(sigma, x);

  NumericVector logitp = as<NumericVector>(wrap(x*A + arma::ones(G)*b));
  NumericVector p2y1__ = plogis(logitp);
  arma::mat p2y1 = arma::mat(as<arma::vec>(p2y1__));  p2y1.reshape(G, J);

  arma::mat log_p2y1 = arma::log(p2y1);
  arma::mat log_p2y0 = arma::log(1-p2y1);

  // -- compute the two weights w0, w1 --
  arma::mat log_p2y (G,J);
  arma::vec log_p2yx(G);
  arma::vec p2yx    (G);
  double    p2y;

  arma::vec tp(G);
  arma::vec w0 = arma::zeros(G);     // final output
  arma::mat w  = arma::zeros(2*G,J); // final output

  int i, j;
  for(i=0; i<N; i++){
```

```
    log_p2yx = log_phi_x;

    for(j=0; j<J; j++){
      if(y(i,j)==1){
        log_p2yx += log_p2y1.col(j);
      }
      else{
        log_p2yx += log_p2y0.col(j);
      }
    }

    p2yx = arma::exp(log_p2yx);
    p2y  = arma::sum(p2yx);
    tp   = p2yx/p2y;

    // -- final output --
    w0 += tp;

    for(j=0; j<J; j++){

      if(y(i,j)==1){
        w(arma::span(0,G-1), j) += tp;
      }
      else{
        w(arma::span(G,2*G-1), j) += tp;
      }

    }

  } // end for i

  // -- return output --
  List output = List::create(Rcpp::Named("w0") = w0,
                             Rcpp::Named("w")  = w
  );
  return output;
}


double obj_func_cpp(arma::mat sigma, arma::mat sigma_hat){
  arma::mat sigma_inv = arma::inv(sigma);
  return arma::accu( sigma_inv % sigma_hat ) + log(arma::det(sigma));
}

// [[Rcpp::export]]
arma::mat calcu_sigma_cmle_cpp(arma::mat sigma_hat, arma::mat sigma0, double tol){
  // This function is modified based on Zhang & Chen (2020).
  // Zhang, S., Chen, Y., & Liu, Y. (2020). An improved stochastic EM algorithm for
  // large-scale full-information item factor analysis. British Journal of
  // Mathematical and Statistical Psychology, 73(1), 44-71.
  arma::mat sigma1 = sigma0;
  arma::mat tmp = sigma0;
  double eps = 1;
  double step = 1;
  while(eps > tol){
    step = 1;
    tmp = arma::inv(sigma0);
```

```
    sigma1 = sigma0 - step * ( - tmp * sigma_hat * tmp + tmp );
    sigma1.diag().ones();
    sigma1 = arma::symmatu(sigma1);
    while(obj_func_cpp(sigma0, sigma_hat) < obj_func_cpp(sigma1, sigma_hat) ||
          min(arma::eig_sym(sigma1)) < 0){
      step *= 0.5;
      sigma1 = sigma0 - step * ( - tmp * sigma_hat * tmp + tmp );
      sigma1.diag().ones();
      sigma1 = arma::symmatu(sigma1);
    }
    eps = obj_func_cpp(sigma0, sigma_hat) - obj_func_cpp(sigma1, sigma_hat);
    // Rprintf("eps= %f\n", eps);
    sigma0 = sigma1;
  }
  return sigma0;
}
```
